# Epidemiological impact and cost‐effectiveness of providing long‐acting pre‐exposure prophylaxis to injectable contraceptive users for HIV prevention in South Africa: a modelling study

**DOI:** 10.1002/jia2.25427

**Published:** 2019-12-19

**Authors:** Marjolein M van Vliet, Cheryl Hendrickson, Brooke E Nichols, Charles AB Boucher, Remco PH Peters, David AMC van de Vijver

**Affiliations:** ^1^ Department of Viroscience Erasmus MC Rotterdam The Netherlands; ^2^ Health Economics and Epidemiology Research Office Department of Internal Medicine School of Clinical Medicine Faculty of Health Sciences University of the Witwatersrand Johannesburg South Africa; ^3^ Department of Global Health Boston University School of Public Health Boston MA USA; ^4^ Department of Medical Microbiology School of Public Health & Primary Care (CAPHRI) Maastricht University Medical Centre Maastricht The Netherlands; ^5^ Anova Health Institute Johannesburg South Africa

**Keywords:** HIV, long‐acting pre‐exposure prophylaxis, injectable contraceptives, cost‐effectiveness, South Africa

## Abstract

**Introduction:**

Although pre‐exposure prophylaxis (PrEP.) is an efficacious HIV prevention strategy, its preventive benefit has not been shown among young women in sub‐Saharan Africa, likely due to non‐adherence. Adherence may be improved with the use of injectable long‐acting PrEP methods currently being developed. We hypothesize that providing long‐acting PrEP to women using injectable contraceptives, the most frequently used contraceptive method in South Africa, could improve adherence to PrEP, result in a reduction of new HIV infections, and be a relatively easy‐to‐reach target population. In this modelling study, we assessed the epidemiological impact and cost‐effectiveness of providing long‐acting PrEP to injectable contraceptive users in Limpopo, South Africa.

**Methods:**

We developed a deterministic mathematical model calibrated to the HIV epidemic in Limpopo. Long‐acting PrEP was provided to 50% of HIV negative injectable contraceptive users in 2018 and scaled‐up over two years. We estimated the number of HIV infections that could be averted by 2030 and the drug price of long‐acting PrEP for which this intervention would be cost‐effective over a time horizon of 40 years, from a healthcare payer perspective. In the base‐case scenario we assumed long‐acting PrEP is 75% effective in preventing HIV infections and 85% of infected individuals are on antiretroviral drug therapy (ART) by 2030. In sensitivity analyses we adjusted PrEP effectiveness and ART coverage. Costs between $519 and $1119 per disability‐adjusted life‐year (DALY) averted were considered potentially cost‐effective, and <$519 as cost‐effective.

**Results:**

Without long‐acting injectable PrEP, 224,000 (interquartile range 176,000 to 271,000) new infections will occur by 2030; use of long‐acting injectable PrEP could prevent 21,000 (17,000 to 26,000) or 9.8% (8.9% to 10.6%) new HIV infections by 2030 (including 6000 (4000 to 7000) in men). Long‐acting PrEP would prevent 34,000 (29,000 to 39,000) or 12,000 (8000 to 15,000) at 75% and 95% ART coverage by 2030 respectively. To be considered potentially cost‐effective the annual long‐acting PrEP drug price should be <$16, and/or ART coverage remains at <85% in 2030.

**Conclusions:**

Providing long‐acting PrEP to injectable contraceptive users in Limpopo is only potentially cost‐effective when long‐acting PrEP drug prices are low. If low prices are not feasible, providing long‐acting PrEP only to women at high risk of HIV infection will become important.

## 1 Introduction

Women in South Africa have a disproportionate risk of HIV‐infection as illustrated by an incidence of 2.5% per year for women aged 15 to 24 years, which is almost five times higher than the incidence in men of the same age. Although this difference between men and women decreases at older ages, the HIV incidence remains higher for women 1. Therefore, South African women might particularly benefit from HIV prevention strategies.

Pre‐exposure prophylaxis (PrEP) is an effective HIV prevention strategy in high‐risk populations with reported risk reductions of 44% to 86% for acquiring HIV infection 2. However, studies of daily oral PrEP in the general population of heterosexual African women have not shown similar efficacy rates. An important reason is low adherence as suggested by drug concentration studies 2, 3, 4.

In the past few years, injectable, long‐acting forms of different antiretroviral drugs have been developed that have the potential for use as long‐acting PrEP 5; the first clinical trial of injectable long‐acting PrEP is ongoing 6. A possible advantage of injectable long‐acting PrEP is that it can reduce the low effectiveness of oral PrEP that is due to poor adherence. Key user groups, including young African women, indicated a preference for long‐acting forms of PrEP compared to daily intake 7, 8, 9. Therefore if long‐acting injectable PrEP were to become available, there may also be greater uptake and longer continuation of use than with daily oral PrEP.

An area that has shown success in long‐acting drug delivery modalities are injectable contraceptives which are used by 25% of South African women aged 15 to 49 years 10. While some observational studies suggested progesterone‐based injectables, particularly depot medroxyprogesterone acetate (DMPA), might increase the risk of HIV infection 11, a recent randomized controlled study did not find an increased risk for HIV for women using DMPA as compared to women using other contraceptive methods 12. However, women using injectable contraceptives might still be an interesting target population for injectable forms of long‐acting PrEP. Combining long‐acting PrEP with injectable contraceptives in a single healthcare visit might improve uptake, adherence and consequently, effectiveness, of long‐acting PrEP 8, 13 and could protect sexually active women against both HIV infection and unintended pregnancies. In addition, a combination of both drugs could decrease overall costs.

Using data on contraceptive use, the HIV epidemic and the ART programme in Limpopo, we aimed to model how many HIV infections could be averted if injectable contraceptive users started using long‐acting PrEP. We also determined the cost at which long‐acting PrEP drugs would be cost‐effective. Limpopo was chosen because we had access to high‐quality data for this part of South Africa through the Anova Health Institute.

## 2 Methods

### 2.1 The model

We developed a mathematical HIV transmission model representing the general HIV epidemic in men and women aged 15 to 49 years old in Limpopo (Figure S1A,B). This compartmental model stratifies disease progression into the acute stage, the chronic stage (sub‐divided in three stages based on CD4 cell count: CD4 level >500, 350 to 500 and 200 to 350 cells/μL), the AIDS stage (CD4 level <200 cells/μL), and in individuals receiving antiretroviral treatment (ART). Each stage of infection has a different duration, infectivity and rate at which infected people start treatment. For example, since most individuals using ART will be virally suppressed, the infectivity when using ART is low (Table 1). We did not consider loss‐to‐follow‐up while using ART. Each stage of infection has a different duration, infectivity and rate at which infected people start treatment. Use of ART reduces the viral load and consequently the infectivity of individuals using ART. Therefore, in our model infectivity when using ART was 87.3%–99.6% lower as compared to the infectivity of individuals with chronic HIV who are not using ART (Table 1). Women are also classified based on contraceptive method use: no contraception, DMPA or any other method (including two monthly injectables). Women can start or stop using injectable contraception or another contraceptive method and can also switch between them. We assume that the use of injectable contraceptives and non‐injectable contraceptives remains stable. In our model, the number of yearly sexual partnerships is equal for men and women (Table 1; Text S1). We assume that the proportion of HIV infected individuals using ART rises from about 66% in 2017 to about 85% in 2030, as predicted by the Thembisa model 14.

**Table 1:** Model parameters

Description	Estimate or range	Reference
Effectiveness long‐acting PrEP	75% (50% to 100%)	15, 16, 17
Average number of sexual partnerships per year after the year 2001^a^	0.53 to 0.97	Model calibration
Increased risk for women to acquire HIV compared to men	2.25 to 4.80	Model calibration
Disease stage infectivity: transmission probability per partnership per year		18; Model calibration
Acute stage	0.28 to 0.68	
Chronic stages	0.081 to 0.15	
AIDS stage	0.28 to 0.62	
On treatment^b^	0.00031 to 0.019	
Mortality rates per year		19
Population	0.029	
Acute HIV	0.102	
Chronic HIV stage	0.102	
AIDS stage	0.633	
On treatment	0.029	Assumption
Disease stages duration		20, 21
Acute stage	10 to 16 weeks	
Chronic stage CD4 >500 cells/μL	0.87 to 1 year	
Chronic CD4 350 to 500 cells/μL	2.9 to 3.1 years	
Chronic CD4 200 to 350 cells/μL	3.6 to 3.9 years	
Rate of getting treatment per year since treatment became available for particular HIV stage^c^		Model calibration
Acute HIV	0.00 to 0.099	
Chronic HIV CD4 >500 cells/μL	0.052 to 0.27	
Chronic HIV CD4 350 to 500 cells/μL	0.052 to 0.27	
Chronic HIV CD4 200 to 350 cells/μL	0.22 to 0.69	
AIDS	0.34 to 0.85	
Rate of getting treatment using long‐acting PrEP	75% after 3 months in acute and chronic stages 100% after 3 months in AIDS stage	Assumption
Contraceptive use in Limpopo (2018 to 2030)^d^		10; Model calibration
Women using DMPA	10.9% (7.0 to 16.2)	
Women using other contraceptives Of which are injectables	38.4% (26.1 to 47.3) 38.7% (28.0 to 43.2)	
New 15 years old per year entering the model	112,000 to 122,000	Model calibration
Costs^e^		22
Yearly cost long‐acting PrEP excluding drug cost ($)	66.91	
Cost of testing negative for HIV per test ($)	4.22	
Cost of testing positive for HIV per test ($)	6.22	
Cost of antiretroviral treatment per year ($)	249.52	
Cost discounting rate per year	3%	
Exchange rate, South African Rand to USD over year 2017	13.86: 1	

a See Table S1 for sexual partnerships over time;

b Infectivity when on treatment depicts an average infectivity of all individuals using anti‐retroviral treatment which includes both virally suppressed and non‐suppressed individuals;

c Values for baseline scenario where 85% of HIV‐infected individuals use antiretroviral therapy (ART) in 2030. Values used after 2017 to obtain different proportions of infected individuals using ART in the sensitivity analysis are depicted in the supplement. Different treatment guidelines over time are taken into account, see supplement;

d Median with minimum and maximum values of simulations in this period;

e See Table S5 for more details.

The model was calibrated to the historic HIV epidemic in Limpopo (2010 to 2017) based on: population size, HIV prevalence for men and women, the proportion of HIV positive individuals receiving ART, and the use of injectable and other forms of contraceptives (see supplement). Using Monte Carlo filtering techniques 23 we accepted 389 of the 250,000 simulations that matched the HIV epidemic in Limpopo. All reported results are the median and interquartile range (IQR) of the accepted simulations. The data for this model came from the publicly available Thembisa model and included data from the years 1985 to 2014 14. No additional data were collected and ethical approval was therefore not required.

### 2.2 Intervention

In Limpopo, about 325,000 HIV negative women aged 15 to 49 years old use injectable contraceptives and are therefore an interesting target group for injectable long‐acting PrEP 10, 14, 24. We assume 50% of this group will use long‐acting PrEP with an effectiveness of 75% 15, 16, 17. Long‐acting PrEP was initiated in 2018 and scaled‐up over two years. Women receiving long‐acting PrEP are tested for HIV infection every three months, the assumed frequency of long‐acting PrEP injections (Table 1).

### 2.3 Epidemiological impact

We estimated the number of HIV infections that could be averted using different effectiveness levels of long‐acting PrEP. The time horizon to determine the epidemiological impact is ten years after a two‐year period of scale‐up (2018 to 2030) In addition, we assessed the impact of different ART coverage levels on the number of new infections and the potential impact of long‐acting PrEP for these different ART scenarios on the number of new infections for the same period.

### 2.4 Cost‐effectiveness

All individuals in our model were assigned a cost and disability‐adjusted life‐year (DALY) based on the stage of HIV‐infection (Table S4). We take a healthcare payer perspective and include costs for HIV testing, ART and long‐acting PrEP. The cost of drugs, laboratory testing, staff and overhead costs were included (Table S5). We varied the drug price of long‐acting PrEP to determine at what price long‐acting PrEP could be cost‐effective. We compared incremental costs and DALYs of long‐acting PrEP use by 50% of injectable contraceptive users, to the scenario of no use of long‐acting PrEP for different effectiveness levels of long‐acting PrEP. The incremental cost‐effectiveness ratios (ICERs) were assessed for a 40‐year time horizon, an estimated lifespan between the average age at HIV infection and the life expectancy in South Africa. Costs and DALYs were discounted at 3% per year. We used the average exchange rate of 2017 to convert South African Rand to US dollars. We compared the median ICER of all accepted simulations to the used country‐level cost‐effectiveness threshold for South Africa, in which ratios below $519 are cost‐effective and ratio's between $519 and $1119 are potentially cost‐effective 25. In addition, we determined scenarios for which at least 90% of our simulations were cost‐effective.

### 2.5 Sensitivity analysis

We estimated the number of averted infections when the absolute uptake of long‐acting PrEP by HIV negative injectable users is lower (25%) or higher (75%) than predicted, if ART use by infected individuals increases to 75% or 95% in 2030 and if the effectiveness of long‐acting PrEP would be lower (50%) or higher (100%) as compared to the assumed 75% effectiveness which is mostly based on animal studies 15, 16, 17. In addition, we did a one‐way sensitivity analysis where we compared the median ICER of our simulations to the used cost‐effectiveness thresholds in which we included ranges for cost and DALY discounting (0% to 5%), the exchange rate (minimum and maximum rate of 2017), costs of ART (±25%), the yearly drug cost of long‐acting PrEP ($0 to $100), the proportion of infected individuals using ART in 2030 (75% to 95%) and long‐acting PrEP effectiveness (50% to 100%). Lastly, we included a scenario where the non‐drug‐related costs for long‐acting PrEP decreased by 30%, since a combination of injectable contraceptives and long‐acting PrEP in a single healthcare visit might reduce the total non‐drug‐related costs for both (Table S5A).

## 3 Results

**Figure 1:**
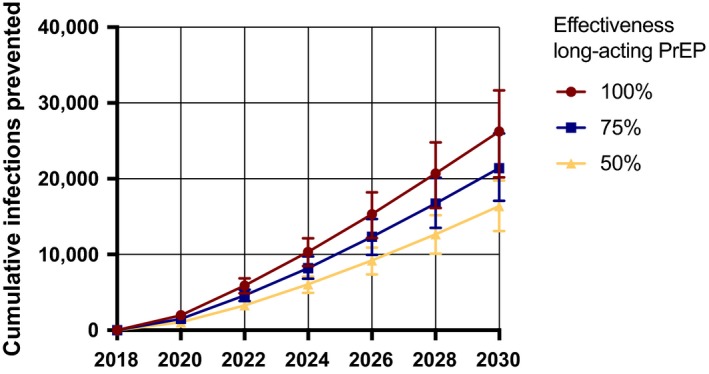
Short‐term epidemiological impact of long‐acting pre‐exposure prophylaxis (PrEP) on the HIV epidemic in Limpopo (2018 to 2030), assuming 50% of injectable contraceptives users use long‐acting PrEP. In the analysis, we assumed an effectiveness of long‐acting PrEP ranging between 50% and 100%. Depicted are the median and interquartile ranges of all accepted simulations.

Long‐acting PrEP, with an effectiveness of 75%, could prevent 22,000 (IQR 18,000 to 27,000) or 9.8% (8.9% to 10.6%) of new HIV infections in Limpopo between 2018 and 2030 (Figure 1) when provided to half of HIV negative injectable contraceptive users. Six thousand (IQR 4000 to 7000) of these infections would be prevented in men showing that long‐acting PrEP is not only beneficial for women (Figure S3). The number of infections potentially averted by long‐acting PrEP increases with long‐acting PrEP effectiveness from 16,000 (IQR 13,000 to 20,000) or 7.5% (IQR 6.9% to 8.2%) to 26,000 (IQR 20,000 to 32,000) or 11.9% (IQR 10.9% to 12.9%) if the effectiveness increases from 50% to 100% respectively. (Figure 1).

### 3.1 Influence of the use of ART and long‐acting PrEP on the number of HIV infections

**Figure 2:**
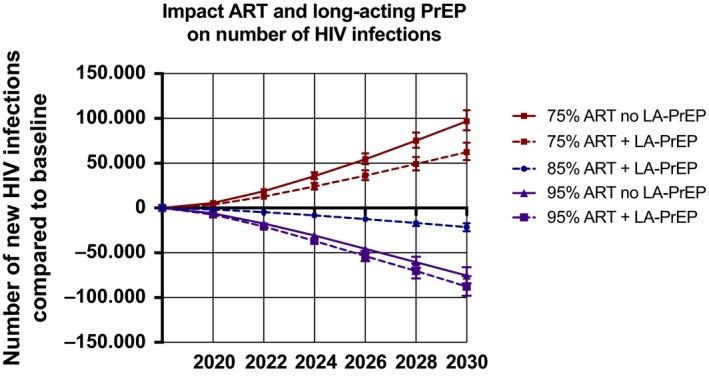
Effects of the coverage with antiretroviral therapy (ART) in the population and long‐acting pre‐exposure prophylaxis (PrEP) provided to half of injectable contraceptive users in Limpopo on the number of new HIV infections in the period 2018 to 2030. Baseline scenario assumes no long‐acting PrEP and 85% of infected individuals using ART by 2030. Effects of lower (75%) and higher (95%) ART coverage in 2030 are depicted as well as the effect of long‐acting PrEP for all three different ART scenarios. Depicted are the median and interquartile ranges of all accepted simulations.

Figure 2 shows the impact of long‐acting PrEP and the proportion of HIV infected individuals using ART on the number of new infections in the period 2018 to 2030. In our baseline scenario where 85% of infected individuals will use ART by 2030 and without long‐acting PrEP, 224,000 (IQR 176,000 to 271,000) new infections will occur. Compared to the scenario where 85% of infected individuals will use ART in 2030, the number of new infections will increase by 97,000 (IQR 87,000 to 109,000) additional infections if only 75% of HIV‐infected receive ART in 2030. Conversely, if 95% of infected individuals will use ART by 2030, 75,000 (IQR 66,000 to 84,000) infections will be prevented compared to the scenario where 85% of infected individuals will use ART in 2030 due to the impact of treatment as prevention. Long‐acting PrEP will prevent more infections if a lower proportion of HIV‐infected individuals receive ART: in our model, the number of averted infections by long‐acting PrEP ranges from 12,000 (IQR 8000 to 15,000) if 95% of infected individuals will use ART by 2030, to 34,000 (IQR 29,000 to 39,000) if 75% of infected individuals will use ART by 2030. Figure S4 shows the impact of long‐acting PrEP if the uptake by injectable contraceptive users is 25% or 75%. The number of infections averted by long‐acting PrEP increases proportional with an increase in the uptake.

### 3.2 Cost‐effectiveness

**Figure 3:**
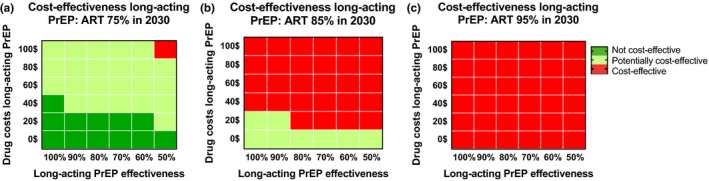
Cost‐effectiveness of providing half of HIV negative injectable contraceptive users in Limpopo with long‐acting pre‐exposure prophylaxis (PrEP) if the proportion of HIV infected individuals using antiretroviral therapy (ART) (**a**) increases to 75% by 2030; (**b**) increases as predicted to 85% by 2030; (**c**) increases to 95% by 2030. A time horizon of 40 years is used. Red represents scenarios not cost‐effective (costs over $1119/DALY), light green represents potentially cost‐effective scenarios (cost between $519–$1119 per DALY) and dark green represents cost‐effective scenarios (cost <$519/DALY). Depicted are the median incremental cost‐effectiveness ratios of all accepted simulations.cting PrEP for all three different ART scenarios. Depicted are the median and interquartile ranges of all accepted simulations.

Figure 3 shows how the cost‐effectiveness of long‐acting PrEP provided to injectable contraceptive users varies with changing effectiveness levels of PrEP and drug costs of long‐acting PrEP using the median ICER of our simulations. By 2030, if 85% of HIV‐positive individuals are on ART and the effectiveness of long‐acting PrEP is 75%, we estimate that yearly drug cost of long‐acting PrEP should be $16 at most to be considered potentially cost‐effective (ICER $1113 IQR $829 to $1696) and will not be considered definitely cost‐effective even if long‐acting PrEP drugs would be free of charge (Figure 3b). Figure S5 shows the cost‐effectiveness results for which at least 90% of our simulations have an ICER below the cost‐effectiveness threshold. If ART use would rise to 85% in 2030, even at a long‐acting PrEP drug price of $0, less than 90% of all simulations are potentially cost‐effective. This is because the use of long‐acting PrEP is also accompanied by non‐drug related costs (Table S5A). A budget impact analyses for the total programme costs in the first one to five years, assuming long‐acting PrEP is 75% effective is provided in Table S6. In a case where there is a slower rise in ART use with 75% of infected individuals on ART by 2030, more infections will be averted by long‐acting PrEP and the cost‐effectiveness increases (Figure 3a). However, if 95% of infected individuals are using ART by 2030, long‐acting PrEP will not be (potentially) cost‐effective (Figure 3c). Cost‐effectiveness results are comparable if 25% or 75% of injectable contraceptive users will use long‐acting PrEP (data not shown).

### 3.3 Sensitivity analysis

**Figure 4:**
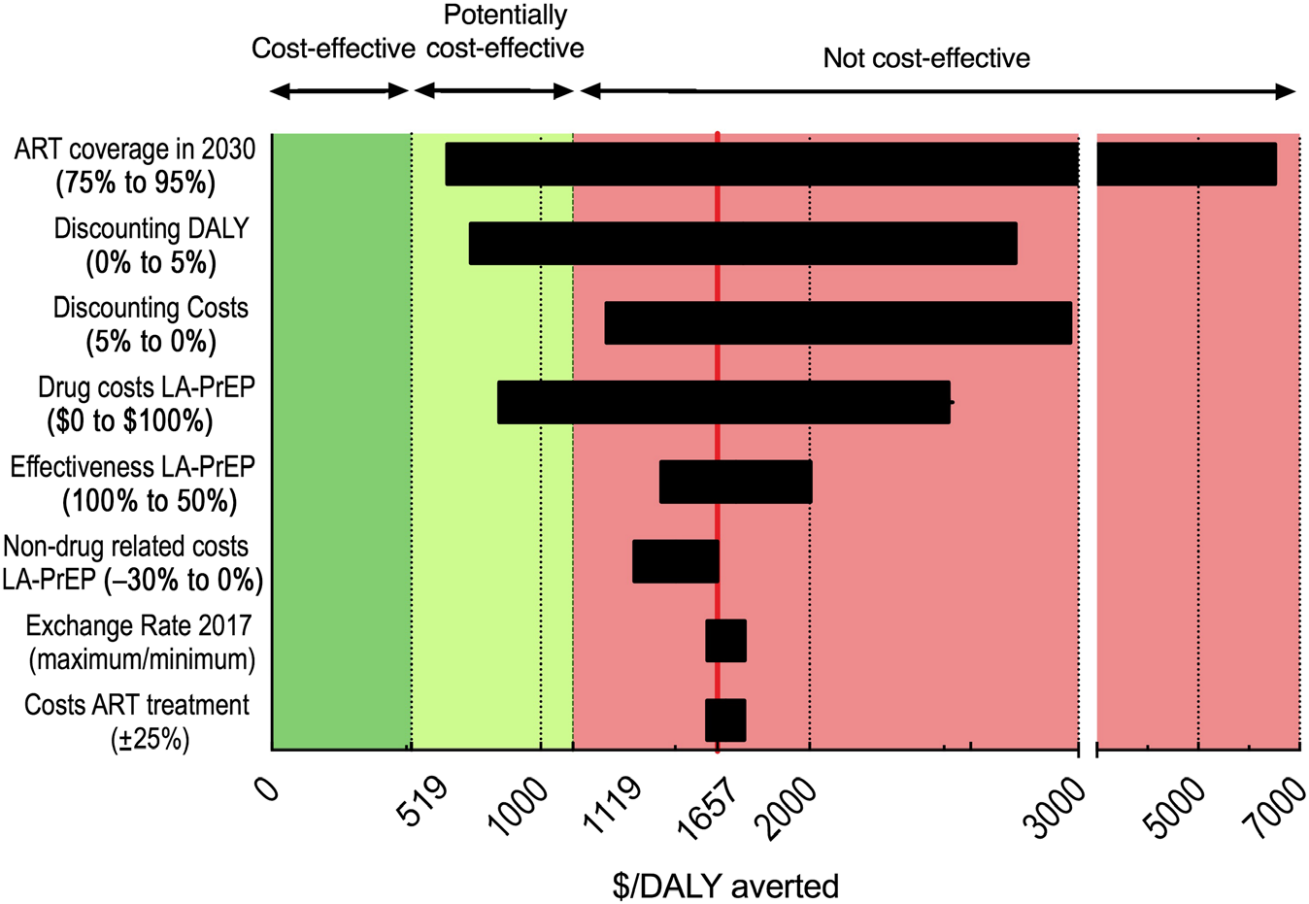
One‐way sensitivity analysis of the incremental cost‐effectiveness of long‐acting pre‐exposure prophylaxis (PrEP) provided to half of HIV negative injectable contraceptive users in Limpopo compared to no long‐acting PrEP over 40 years. At baseline, the proportion of infected individuals using antiretroviral therapy (ART) will rise as predicted to 85% in 2030, long‐acting PrEP is 75% effective, long‐acting PrEP drug costs $50 per year and non‐drug related costs for long‐acting PrEP are $66.91. Bars show the change in cost‐effectiveness if the value of the corresponding parameter is replaced by the value in parentheses. Red represents scenarios not cost‐effective (costs over $1119/DALY), light green represents potentially cost‐effective scenarios (cost between $519 and $1119 per DALY) and dark green represents cost‐effective scenarios (cost <$519/DALY). Depicted are the median incremental cost‐effectiveness ratios of all accepted simulations. Baseline scenario cost $1657 per DALY. DALY, disability‐adjusted life‐year, LA‐PrEP, long‐acting pre‐exposure prophylaxis.

The cost per DALY averted of long‐acting PrEP provided to injectable contraceptive users is most sensitive to ART use (Figure 4) and ranges from $652 to $6500/DALY for an ART coverage of 75% or 95% in 2030 respectively. Other important parameters affecting the cost‐effectiveness of long‐acting PrEP are yearly DALY and costs discounting rates and yearly long‐acting PrEP drug cost; long‐acting PrEP is not (potentially) cost‐effective for most scenarios.

## 4 Discussion

In this study, we investigated the impact and cost‐effectiveness of long‐acting PrEP provided to HIV negative injectable contraceptive users in Limpopo. Our model predicts that 22,000 or 9.8% of new infections could be prevented between 2018 and 2030 if half of HIV negative injectable contraceptive users (11% of women aged 15 to 49 years) use long‐acting PrEP. Long‐acting PrEP drug price should cost $16 or less to be potentially cost‐effective.

The cost‐effectiveness results are highly sensitive to ART coverage. If ART coverage increases, the preventative impact and cost‐effectiveness of long‐acting PrEP decrease. However, increasing ART use will prevent more infections compared to increasing uptake of long‐acting PrEP by injectable contraceptive users. Therefore, efforts should be made to identify HIV infections in a timely manner, to increase ART use and to retain infected individuals in care to make sure they are virally suppressed, in line with the current WHO guidelines 26. Long‐acting PrEP may then also be most cost‐effective sooner, rather than later, while ART coverage remains relatively low (±66% in Limpopo in 2017) 14. Long‐acting PrEP provided to injectable contraceptive users was not found to be cost‐saving, even at a long‐acting PrEP drug price of $0. Additionally, less than 90% of accepted simulations had an ICER below the potentially cost‐effectiveness threshold in our baseline scenario, when long‐acting PrEP drugs were free (Figure S5). This is because long‐acting PrEP use is also accompanied by non‐drug‐related costs (Table S5A).

In this study, we show that providing injectable contraceptive users with long‐acting PrEP is only cost‐effective when long‐acting drug prices are low or if the HIV incidence remains relatively high. Yearly drug cost of oral PrEP for women in South Africa is estimated to be about $61 per person 22. However, when newly developed, it is likely that drug prices for injectable long‐acting PrEP will even be higher. So, even though the long‐acting PrEP drug price is not known yet, a yearly drug costs <$16 per person is not expected. If low prices are not feasible and ART coverage increases as predicted, it will become important to provide long‐acting PrEP only to populations with higher incidence rates. In that case, long‐acting PrEP should be targeted at women who are at high risk of HIV infection. In practice, women using injectable contraceptives might have a higher HIV risk compared to women in general, because they will be sexually active and might have fewer incentives to use condoms. However, differences in risk behaviour between injectable contraceptive users and women not using injectable contraceptives were not assessed in our model, since the extent of differences in sexual risk behaviour are not known.

We assumed that half of HIV negative injectable contraceptive users will use long‐acting PrEP and injectable contraceptive use will remain stable. Data about the potential uptake of PrEP by women differ widely and the uptake will also be influenced by the effectiveness, safety, costs and side effects of long‐acting PrEP as well as implementation strategies 8, 13, 27, 28, 29. However, in populations using injectable contraceptives, acceptability of injectable long‐acting PrEP will probably be high and this combination could increase the use of both drugs 8, 13. If women who are using no or other contraceptive methods switch to injectable contraceptives with long‐acting PrEP, the number of infections potentially averted by long‐acting PrEP would increase. However, a change in uptake of long‐acting PrEP had no effect on the cost‐effectiveness. In practice, women not using injectable contraceptives could also start long‐acting PrEP when available. In our model, the risk for HIV is comparable for women using injectable contraceptives and the general population of women. Therefore, cost‐effectiveness results would be comparable if women not using injectable contraceptives would start long‐acting PrEP.

Use of injectable contraceptives by sexually active women in South Africa is highest for women aged 15 to 34 10. Therefore, providing long‐acting PrEP to injectable contraceptive users may disproportionately contribute to HIV prevention among younger women, who have a higher HIV incidence 1. As such, our results are likely an underestimate of the total benefits of PrEP targeted to this population.

Combining long‐acting PrEP with injectable contraceptives in a single health care visit could not only increase uptake and adherence for both drugs, but could also decrease overall costs. This cost reduction would improve cost‐effectiveness of long‐acting PrEP, as shown in our sensitivity analyses. A possible drawback of combining both drugs in a single healthcare visit could be a mismatch in dosing regimens. Development of long‐acting PrEP with different available dosing regimens is therefore desirable. However, if long‐acting PrEP has to be administered more frequently, long‐acting PrEP costs will increase. Additionally, uptake of long‐acting PrEP might increase if both injections could be combined in a single shot.

Effectiveness of most antiretroviral drugs and injectable contraceptives is not affected when combined together 30. However, we recommend to study interactions between long‐acting PrEP and injectable contraceptives more extensively as well as other possible side effects. Side effects of long‐acting PrEP could decrease its cost‐effectiveness. Recently, concerns about safety during conception when using dolutegravir 31, an analogue of cabotegravir which is currently investigated as long‐acting PrEP (6), have been raised. These concerns could delay the development of long‐acting PrEP suitable for women of childbearing age. However, dolutegravir can be used by women who are using effective contraceptives 31, like injectable contraceptive users.

Our model has several strengths. First, we accounted for the population‐level effect of PrEP, meaning PrEP is not only beneficial for individuals using PrEP, but also for (future) partners of protected individuals. Second, we take into account the newest WHO guideline regarding ART and show how this could impact the HIV epidemic as well as the potential impact of long‐acting PrEP. Lastly, we calibrated our model to the well‐described HIV epidemic in Limpopo and accurate data about the ART programme in this region.

Our model has several limitations. First, we do not consider a possible increase in resistance against antiretroviral drugs using long‐acting PrEP. Previous modelling studies estimated HIV drug resistance resulting from PrEP will probably be limited 32. However, risk of resistance is probably higher for long‐acting PrEP since these formulations are associated with prolonged suboptimal drug levels that could facilitate the emergence of drug resistance 33. An oral PrEP ‘tail’ may therefore be required, where oral PrEP is taken for several months after the discontinuation of injectable PrEP, and the effectiveness and adherence to this “tail” are unknown. Second, all individuals in our model have the same number of sexual partnerships in each model simulation, and no stratification by age was considered. Since long‐acting PrEP targeted at women with a higher HIV incidence improves its cost‐effectiveness, providing long‐acting PrEP to young women and other high‐risk populations will improve cost‐effectiveness of long‐acting PrEP. However, there is no reliable data about sexual risk behaviour in the population for different age categories and by use of contraceptive method, and identifying highly sexually active individuals is difficult. Lastly, the future uptake of long‐acting PrEP may be much lower than the 50% we assumed in our base‐case scenario. Reducing the coverage of long‐acting PrEP will, however, result in a comparable cost‐effectiveness ratio.

Other studies have modelled the effect of oral or injectable PrEP provided to the general population 34, 35, 36, 37, 38, 39 or to women 36, 40, 41, 42 in an African setting, where 42 especially modelled cost‐effectiveness of methods combining HIV and pregnancy prevention. However, model input parameters and outcome measures often differ between studies, complicating comparison. For example, most studies were performed before the current WHO guidelines regarding start of ART became effective and time horizons chosen range between 10 years to a lifetime. Most studies show PrEP could be cost‐effective in an African setting when provided to individuals at high risk of HIV. Quaife *et al* 42 shows different scenarios for preventing pregnancies and HIV infections using multi‐purpose prevention technologies in South Africa. They show this would only be cost‐effective for women aged 16 to 24, but not for women aged 25 to 49 because of higher incidence rates for younger women. Contrary to our model, as Quaife *et al* used a static model, they could not consider the secondary infections that can be prevented on a population level due to use of PrEP. In line with our results, multiple studies show cost‐effectiveness of PrEP decreases rapidly if ART use increases and more infections can be averted by increasing ART compared to PrEP 35, 36, 38, 39, 40. Lastly, when comparing the different studies, it is important to note that South Africa does not have a generally accepted cost‐effectiveness threshold. As a consequence, the particular threshold that is used should be considered when comparing different cost‐effectiveness studies.

## 5 Conclusions

In summary, long‐acting injectable forms of PrEP are under development and could improve continuation of PrEP and reduce effectiveness problems due to low adherence, which have been observed in randomized controlled trials studying oral PrEP in African women 2, 3, 4. Injectable contraceptive users are an interesting target group if injectable long‐acting PrEP becomes available. This is one potential way to identify sexually active women at risk of HIV that find injections tolerable. The combination of both injections in a single healthcare visit could decrease costs and improve uptake and adherence for both drugs.

However, long‐acting PrEP provided to injectable contraceptive users in Limpopo could only be potentially cost‐effective if drug prices of long‐acting PrEP will be low and challenges with increasing ART use by infected individuals remain. To improve cost‐effectiveness of long‐acting PrEP, it is desirable to identify and target subpopulations with high HIV incidences who are therefore at highest risk of HIV infection.

## Competing interest

DAMCvdV and CABB report grants from Gilead Sciences, MSD, ViiV health care and Janssen outside the submitted work. DAMCvdV reports advisory work to ViiV outside the submitted work. All other authors do not report a conflict of interest.

## Authors' contributions

CABB, RPHP, DAMCvdV conceived and designed the study. MvV and DAMCvdV developed and programmed the mathematical model. MvV, CH, BENN and RPHP performed the data analysis. MvV and DAMCvdV wrote the first draft of the manuscript. MvV, CH, BENN, CABB, RPHP and DAMCvdV contributed to writing of the manuscript. MvV, CH, BENN, CABB, RPHP and DAMCvdV read and approved the final manuscript.

## Supporting information


**Figure S1.** Model structure. **A**, Men. **B**, Women.

**Figure S2.** The model is calibrated to (**A**) The population size in Limpopo (**B**) Proportion HIV‐infected individuals (15 to 49 years) using antiretroviral treatment (ART) in Limpopo (**C**) HIV prevalence in men (15 to 49 years) in Limpopo and (**D**) HIV prevalence in women (15 to 49 years) in Limpopo. The HIV prevalence in men and women and the proportion of HIV‐infected individuals using ART are calibrated to data from the Thembisa model which is based on historic data. Depicted are the median (black line) with the minimum and maximum values of all accepted simulations in orange.

**Figure S3.** Epidemiological impact of long‐acting pre‐exposure prophylaxis (PrEP) with an effectiveness of 75% on the HIV epidemic in Limpopo, assuming 50% of injectable contraceptives users use long‐acting PrEP and 85% of infected individuals uses antiretroviral therapy (ART) by 2030. Total prevented infections and infections prevented separated by men and women are depicted. Prevented infections in men are an indirect result of long‐acting PrEP use by women. Depicted are the median and interquartile ranges of all accepted simulations.

**Figure S4.** Effects of the coverage with antiretroviral therapy [23] in the population and long‐acting pre‐exposure prophylaxis (PrEP) targeted to injectable contraceptive users in Limpopo on the number of new HIV infections in the period 2018 to 2030. Baseline scenario assumes no long‐acting PrEP and 85% of infected individuals using ART by 2030. Effects of lower (75%) and higher (95%) ART coverage in 2030 are depicted as well as the effect of long‐acting PrEP for all three different ART scenarios. (A) 25% of HIV‐negative injectable users use long‐acting PrEP (B) 75% of HIV‐negative injectable users use long‐acting PrEP. Depicted are the median and interquartile ranges of all accepted simulations. LA‐PrEP = long‐acting pre‐exposure prophylaxis

**Figure S5.** Cost‐effectiveness of providing half of HIV negative injectable contraceptive users in Limpopo with long‐acting pre‐exposure prophylaxis (PrEP.) if the proportion of HIV infected individuals using antiretroviral therapy (ART) (**a**) increases to 75% by 2030; (**b**) increases as predicted to 85% by 2030; (**c**) increases to 95% by 2030. A time horizon of 40 years is used. Red represents scenarios not cost‐effective (costs over $1119/DALY), light green represents potentially cost‐effective scenarios (cost between $519–$1119 per DALY) and dark green represents cost‐effective scenarios (cost <$519/DALY). To be considered (potentially) cost‐effective, at least 90% of accepted simulations have an incremental cost‐effectiveness ratio below the (potentially) cost‐effectiveness threshold.

**Text S1.** Full model description and equations
**Table S1.** Number of sexual partnerships per year for different periods in time
**Table S2.** **A**, Calibrated variables for the use of different contraceptive methods in the period before scale‐up of long‐acting pre‐exposure prophylaxis (PrEP). **B**, Variables after scale‐up period of long‐acting pre‐exposure prophylaxis (PrEP). Values are adapted to obtain calibrated proportions of injectable contraceptive users on long‐acting PrEP
**Table S3.** Range of values used for rates of getting treatment after 2017. The rate of starting treatment before 2018 will result in an overall proportion of 85% of HIV infected individuals that will be using treatment in 2030. The HIV treatment rates in 2018 and after were varied equally for all disease stages to obtain 75% or 95% of HIV‐infected individuals using ART in 2030
**Table S4.** Assumed disability weightings for DALYs
**Table S5.** **A**, Costs for HIV testing, antiretroviral treatment and long‐acting pre‐exposure prophylaxis (PrEP) in Limpopo [15]. **B**, Overview used costs
**Table S6.** Variables used to calibrate and accept simulations using Monte Carlo filtering techniques. 348 simulations were accepted from 490,000 total simulations run
**Table S7.** Budget impact of providing half of HIV negative injectable contraceptive users with long‐acting PrEP, assuming our baseline scenario with 85% ART coverage in 2030 and long‐acting PrEP effectiveness of 75%. Program costs are depicted in million dollars. Drug costs of long‐acting PrEP are ranged between $0 to $100 per person per year, including $16, the maximum drug price for which long‐acting PrEP was found to be potentially cost‐effective. Non‐drug related costs are $66.91, see Table S5A. Time horizons range between one to five years after full scale up. PrEP, pre‐exposure prophylaxes
