# Supplementary figures and images for: Epidemiological impact and cost‐effectiveness of providing long‐acting pre‐exposure prophylaxis to injectable contraceptive users for HIV prevention in South Africa: a modelling study
Source: J Int AIDS Soc. 2019 Dec 19;22(12):e25427. doi: 10.1002/jia2.25427 (PMC6922023; doi:10.1002/jia2.25427)

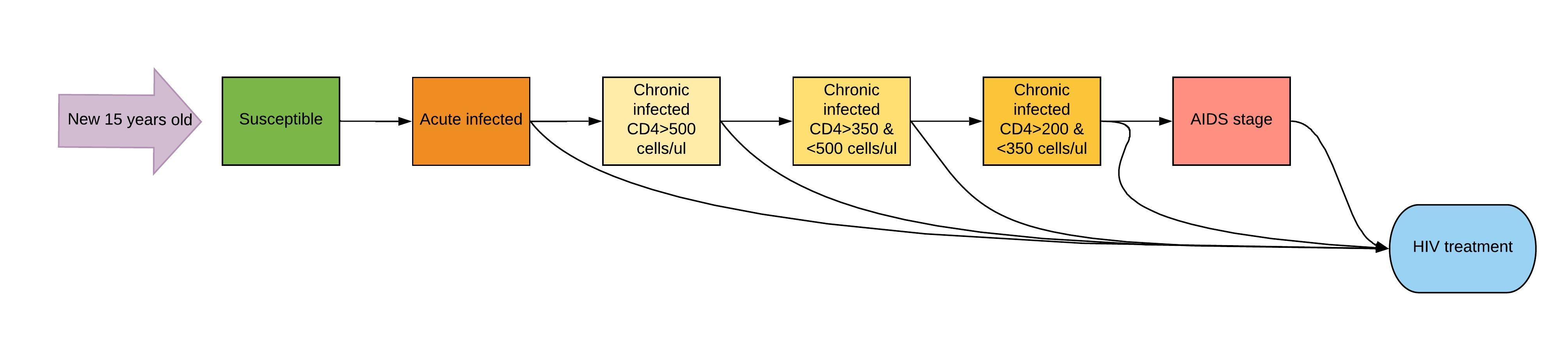

Supplement: Supplementary file 1 — Figure S1. Model structure. A, Men. B, Women. [file JIA2-22-e25427-s001.jpeg]

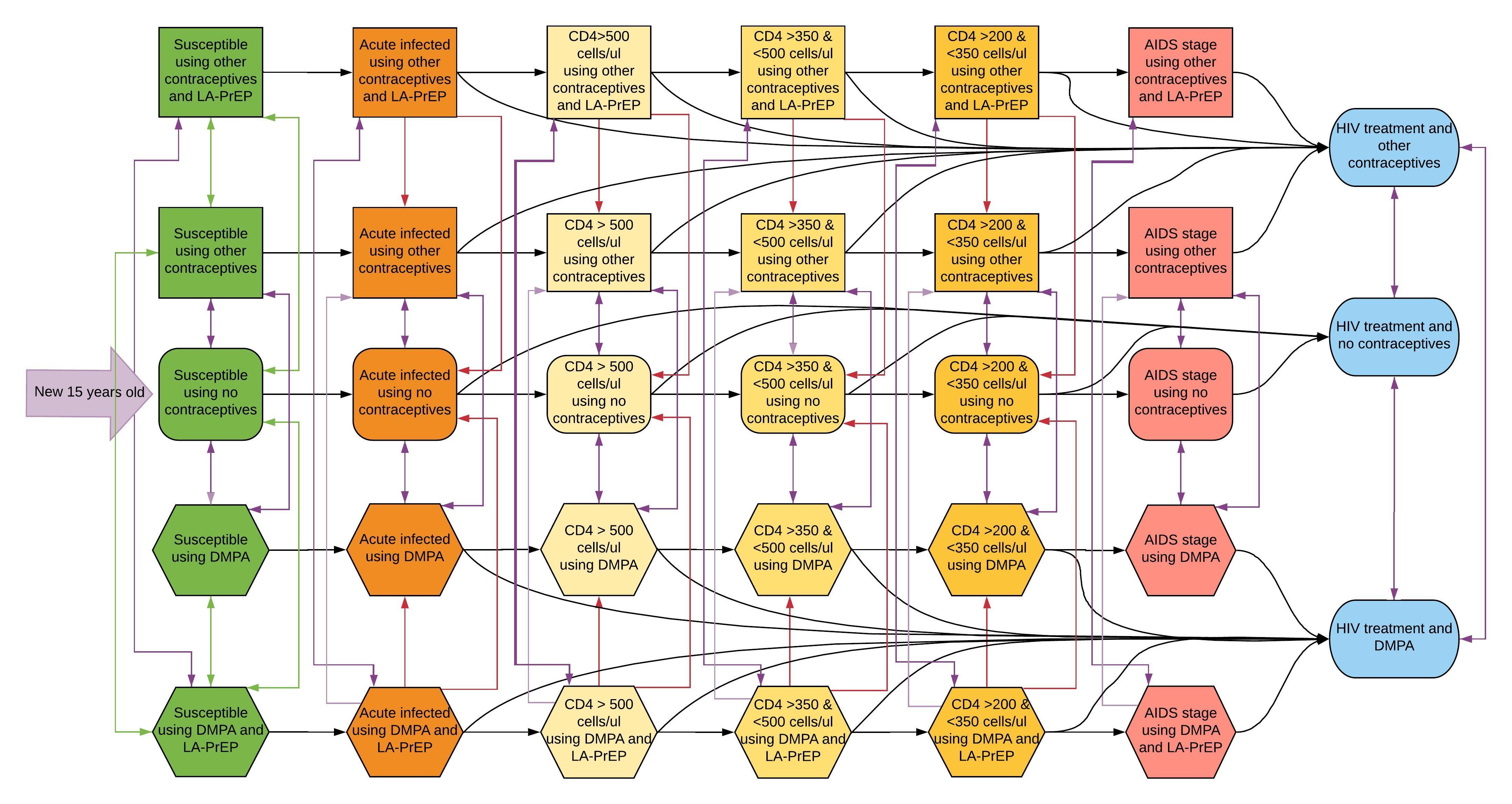

Supplement: Supplementary file 2 — Figure S2. The model is calibrated to (A) The population size in Limpopo (B) Proportion HIV‐infected individuals (15 to 49 years) using antiretroviral treatment (ART) in Limpopo (C) HIV prevalence in men (15 to 49 years) in Limpopo and (D) HIV prevalence in women (15 to 49 years) in Limpopo. The HIV prevalence in men and women and the proportion of HIV‐infected individuals using ART are calibrated to data from the Thembisa model which is based on historic data. Depicted are the median (black line) with the minimum and maximum values of all accepted simulations in orange. [file JIA2-22-e25427-s002.jpeg]

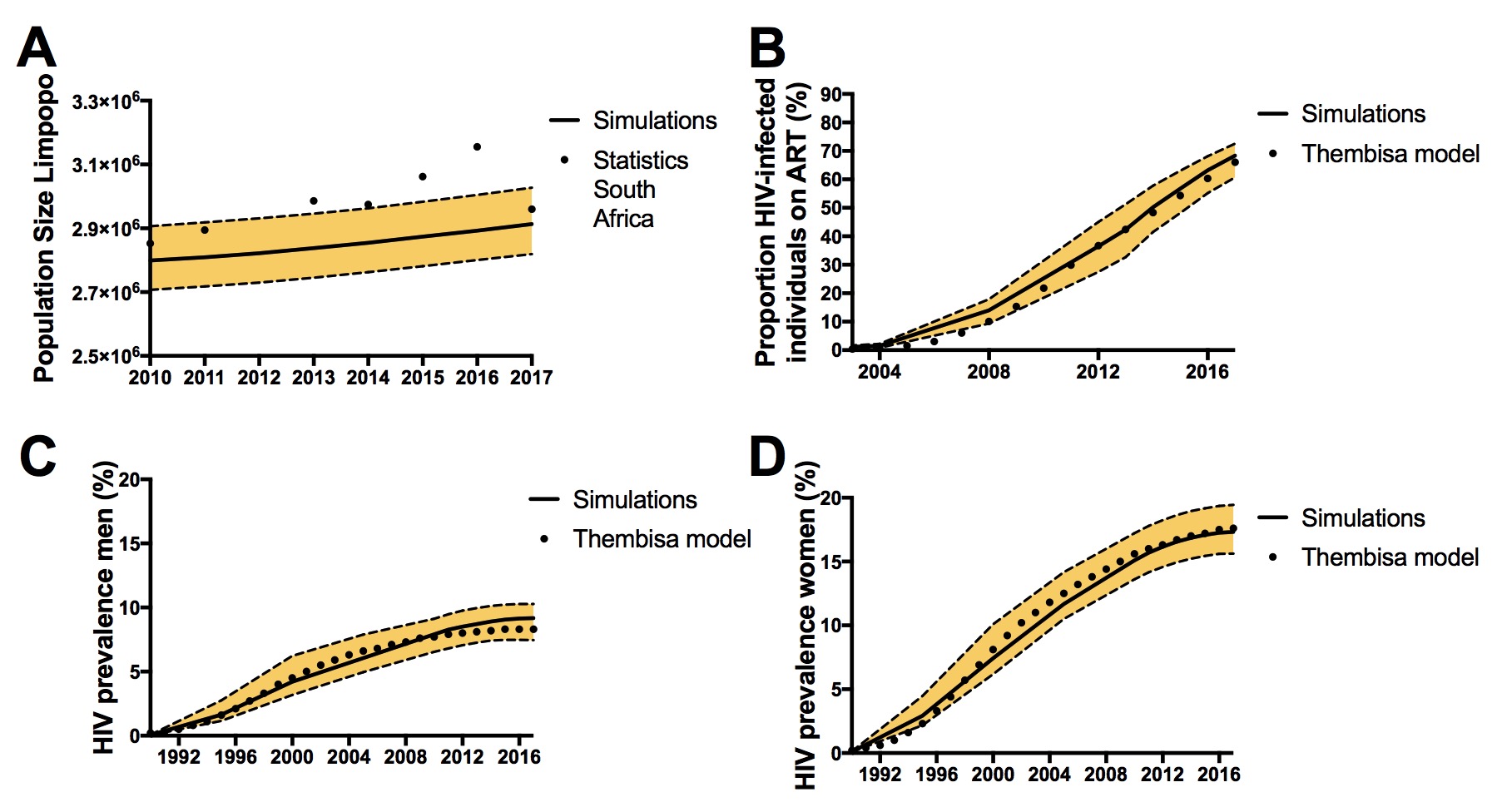

Supplement: Supplementary file 3 [file JIA2-22-e25427-s003.jpg]

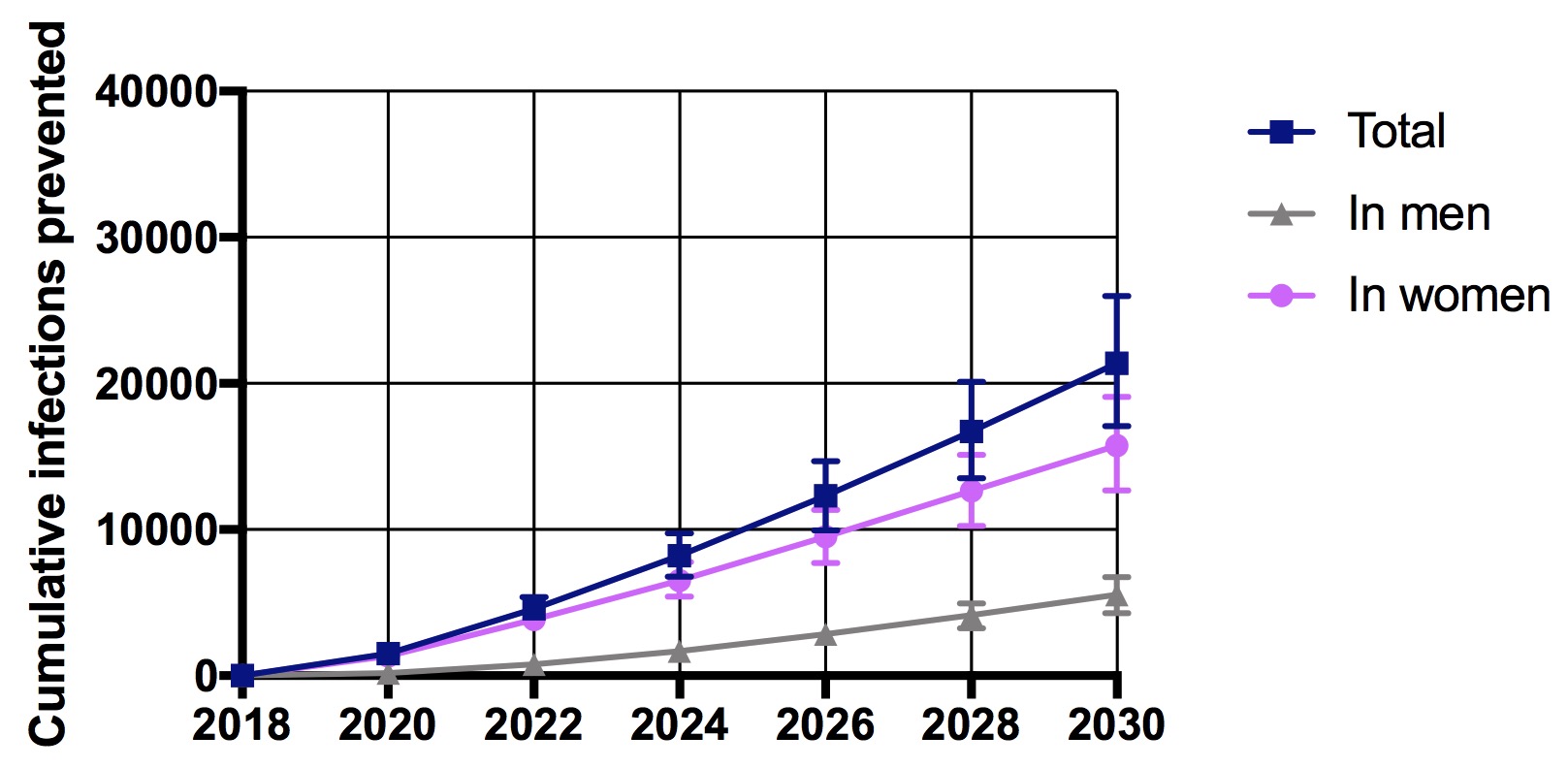

Supplement: Supplementary file 4 — Figure S3. Epidemiological impact of long‐acting pre‐exposure prophylaxis (PrEP) with an effectiveness of 75% on the HIV epidemic in Limpopo, assuming 50% of injectable contraceptives users use long‐acting PrEP and 85% of infected individuals uses antiretroviral therapy (ART) by 2030. Total prevented infections and infections prevented separated by men and women are depicted. Prevented infections in men are an indirect result of long‐acting PrEP use by women. Depicted are the median and interquartile ranges of all accepted simulations. [file JIA2-22-e25427-s004.jpg]

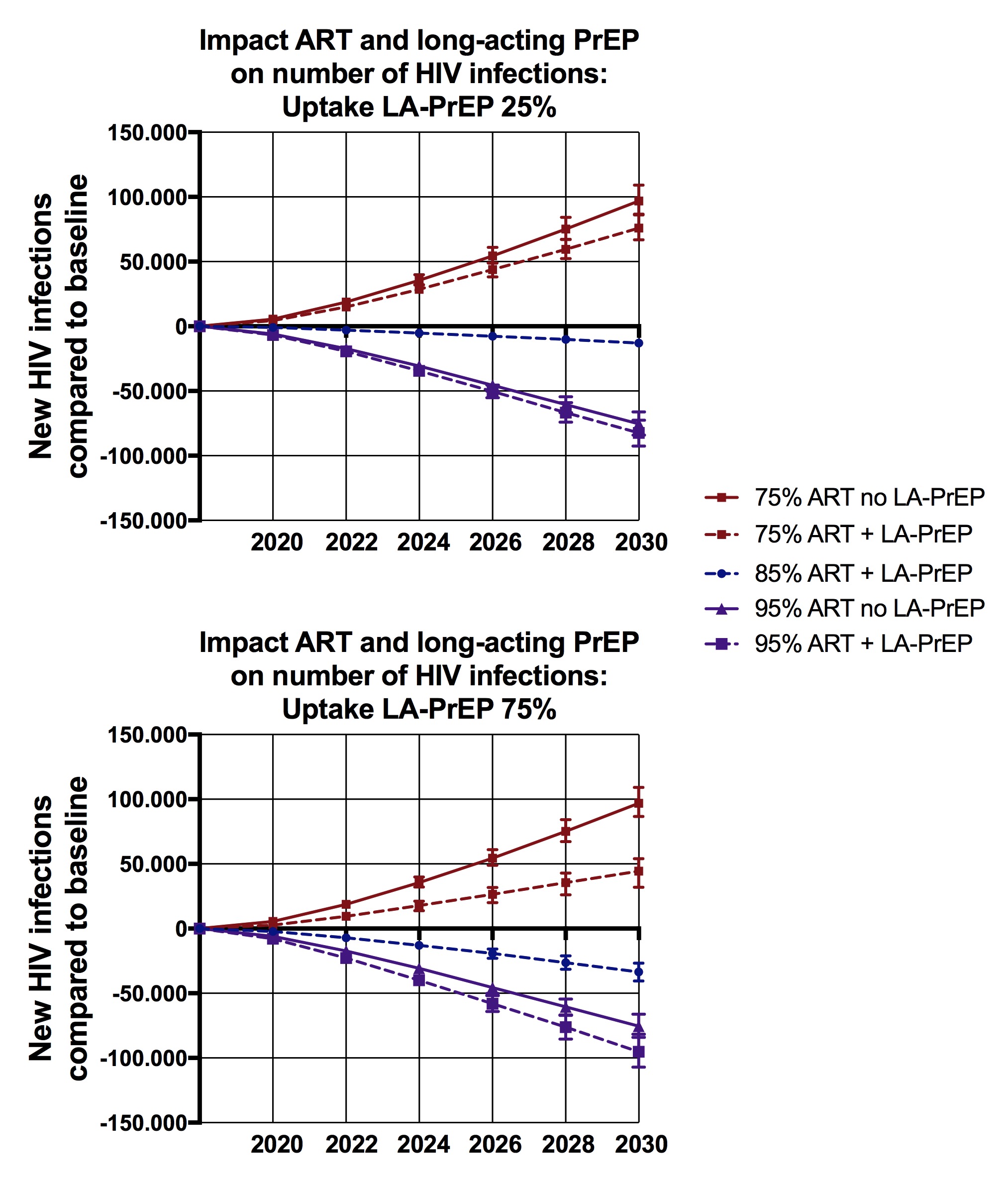

Supplement: Supplementary file 5 — Figure S4. Effects of the coverage with antiretroviral therapy [23] in the population and long‐acting pre‐exposure prophylaxis (PrEP) targeted to injectable contraceptive users in Limpopo on the number of new HIV infections in the period 2018 to 2030. Baseline scenario assumes no long‐acting PrEP and 85% of infected individuals using ART by 2030. Effects of lower (75%) and higher (95%) ART coverage in 2030 are depicted as well as the effect of long‐acting PrEP for all three different ART scenarios. (A) 25% of HIV‐negative injectable users use long‐acting PrEP (B) 75% of HIV‐negative injectable users use long‐acting PrEP. Depicted are the median and interquartile ranges of all accepted simulations. LA‐PrEP = long‐acting pre‐exposure prophylaxis [file JIA2-22-e25427-s005.jpg]

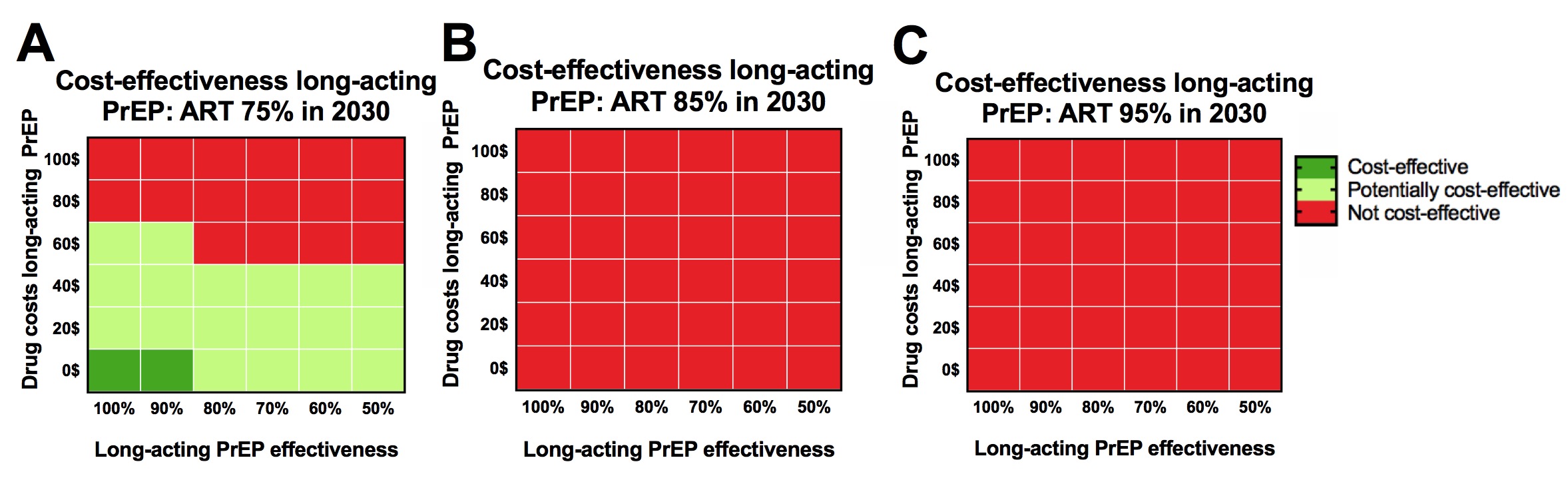

Supplement: Supplementary file 6 — Figure S5. Cost‐effectiveness of providing half of HIV negative injectable contraceptive users in Limpopo with long‐acting pre‐exposure prophylaxis (PrEP.) if the proportion of HIV infected individuals using antiretroviral therapy (ART) (a) increases to 75% by 2030; (b) increases as predicted to 85% by 2030; (c) increases to 95% by 2030. A time horizon of 40 years is used. Red represents scenarios not cost‐effective (costs over $1119/DALY), light green represents potentially cost‐effective scenarios (cost between $519–$1119 per DALY) and dark green represents cost‐effective scenarios (cost <$519/DALY). To be considered (potentially) cost‐effective, at least 90% of accepted simulations have an incremental cost‐effectiveness ratio below the (potentially) cost‐effectiveness threshold. [file JIA2-22-e25427-s006.jpg]
