# Supplementary material for: Epidemiological impact and cost‐effectiveness of providing long‐acting pre‐exposure prophylaxis to injectable contraceptive users for HIV prevention in South Africa: a modelling study
Source: J Int AIDS Soc. 2019 Dec 19;22(12):e25427. doi: 10.1002/jia2.25427 (PMC6922023; doi:10.1002/jia2.25427)
Supplement: Supplementary file 7 — Text S1. Full model description and equations Table S1. Number of sexual partnerships per year for different periods in time Table S2. A, Calibrated variables for the use of different contraceptive methods in the period before scale‐up of long‐acting pre‐exposure prophylaxis (PrEP). B, Variables after scale‐up period of long‐acting pre‐exposure prophylaxis (PrEP). Values are adapted to obtain calibrated proportions of injectable contraceptive users on long‐acting PrEP Table S3. Range of values used for rates of getting treatment after 2017. The rate of starting treatment before 2018 will result in an overall proportion of 85% of HIV infected individuals that will be using treatment in 2030. The HIV treatment rates in 2018 and after were varied equally for all disease stages to obtain 75% or 95% of HIV‐infected individuals using ART in 2030 Table S4. Assumed disability weightings for DALYs Table S5. A, Costs for HIV testing, antiretroviral treatment and long‐acting pre‐exposure prophylaxis (PrEP) in Limpopo [15]. B, Overview used costs Table S6. Variables used to calibrate and accept simulations using Monte Carlo filtering techniques. 348 simulations were accepted from 490,000 total simulations run Table S7. Budget impact of providing half of HIV negative injectable contraceptive users with long‐acting PrEP, assuming our baseline scenario with 85% ART coverage in 2030 and long‐acting PrEP effectiveness of 75%. Program costs are depicted in million dollars. Drug costs of long‐acting PrEP are ranged between $0 to $100 per person per year, including $16, the maximum drug price for which long‐acting PrEP was found to be potentially cost‐effective. Non‐drug related costs are $66.91, see Table S5A. Time horizons range between one to five years after full scale up. PrEP, pre‐exposure prophylaxes [file JIA2-22-e25427-s007.docx]

**Supplementary data for** *Epidemiological impact and cost-effectiveness of long-acting pre-exposure prophylaxis combined with injectable contraceptives for HIV prevention in South Africa: A modeling study*

**Figure S1A. Model Structure Men**

*Described in Text S1.*


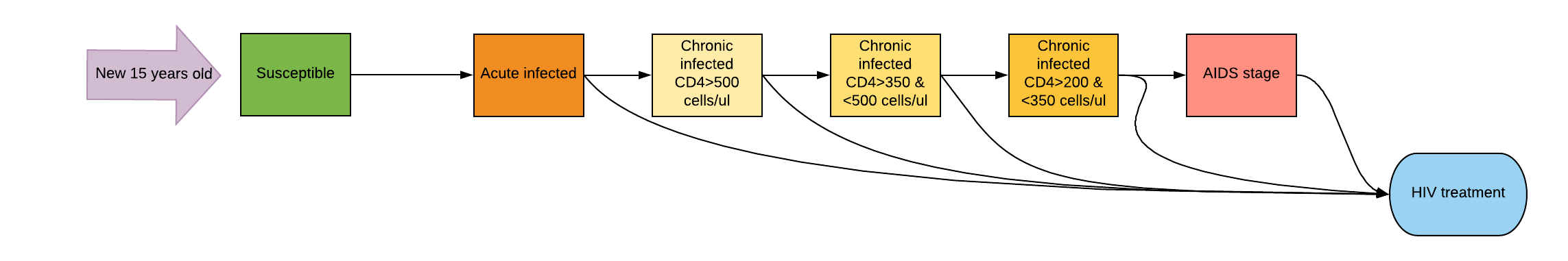


**Figure S1B. Model Structure Women**

Described in *Text S1*.


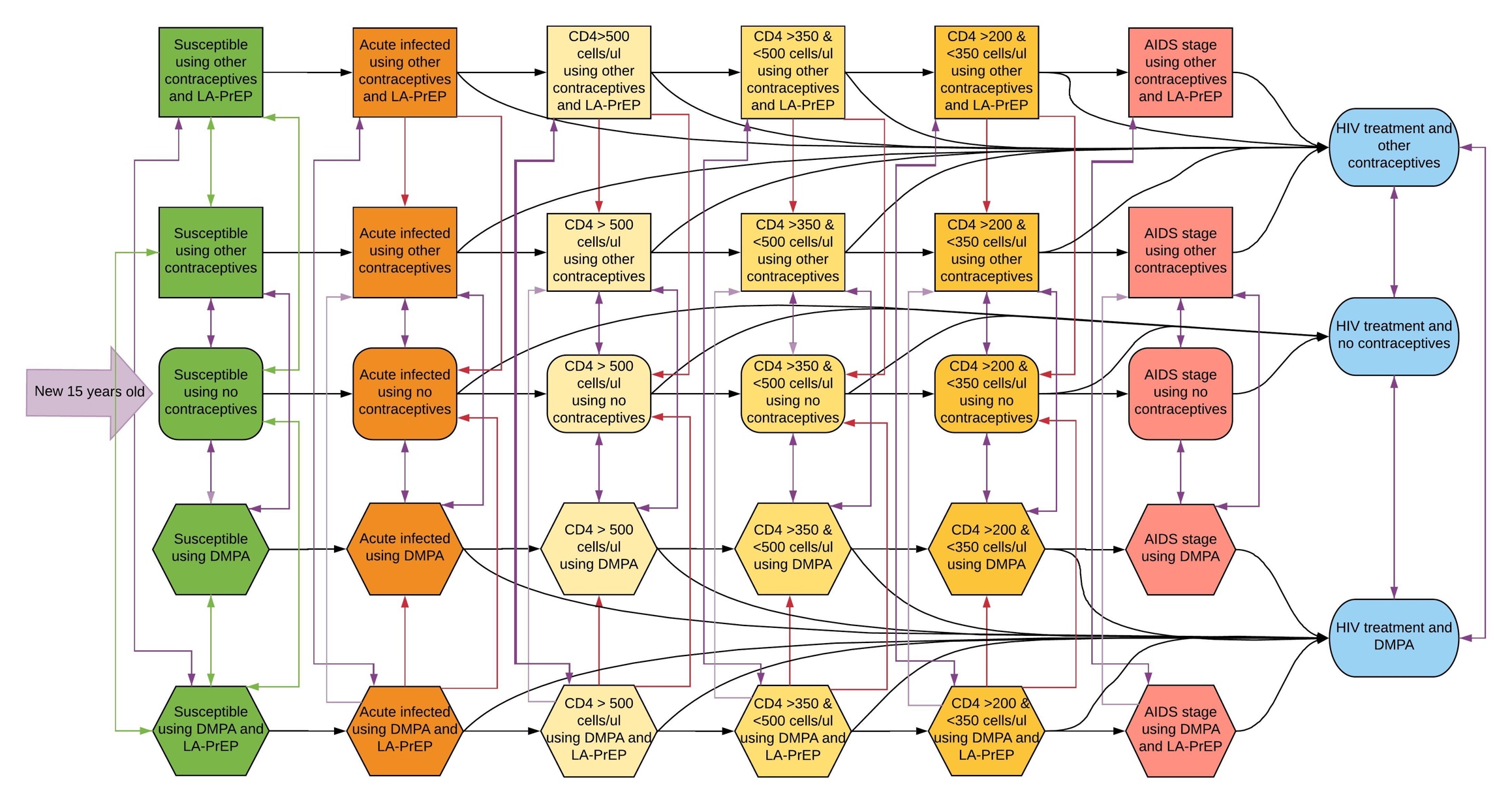


**Text S1. Full model description and equations**

The model is seeded in 1975 with one infected woman and one infected man. The state variables and HIV transmission equations for the model are shown below. For women, these equations also include the changes in the use of contraceptives. Women in this model can use no contraceptives, depot medroxyprogesterone acetate (DMPA.) *(D)** or ‘other contraceptives’ *(C).* Women can start or discontinue the use of contraceptives and can switch between them. Contraceptives other than DMPA include the use of two monthly injectables. Women using DMPA or other injectables can start using long-acting PrEP as long as they are not infected with HIV.

The model included five HIV infection stages *k*: stage 1 is the acute stage, stage 2 is the chronic stage with CD4 >500 cells per μl, stage 3 is the chronic stage with CD4 >350 and <500 cells per μl, stage 4 is the chronic stage with CD4>200 and <350 cells per μl and stage 5 is the AIDS stage with CD4 <200 cells per μl. Infected individuals (*I*) are in class *I* when infected and not yet on treatment, in class *P* when infected while using long-acting PrEP and not yet on treatment, and class *RX* when they are using antiretroviral treatment (ART).

All groups have their own mortality rate (μ) depending on HIV stage (table 1, not depicted in figure S1). The force of infection ($\lambda$) describes the rate by which susceptible individuals become infected with HIV. The HIV progression rate (γ) represents the duration of the different HIV stages (table 1). The rate by which infected individuals without the use of long-acting PrEP start HIV treatment (RX_K_) is calibrated in the model and differs per HIV stage (table 1). Women who use long-acting PrEP are tested for HIV every three months, and 75% starts treatment after HIV diagnosis in our model, the other 25% only discontinues long-acting PrEP. For women using long-acting PrEP who are in the AIDS stage, all patients start treatment after diagnosis because of ethical reasons.

*DMPA and other injectable contraceptives are modelled separately, since the model was first developed with a higher risk of becoming infected when using DMPA [1]. However, in light of the ECHO trial [2], the model was calibrated again without this increased risk. So, all women in our model have the same risk of becoming HIV infected, irrespective of contraception use.

*State variables*

SM = Susceptible men

${IM}_{k}^{I}$ = HIV infected men, k = 1..5

${IM}^{RX}$ = HIV infected men receiving treatment

SF = Susceptible women not using contraceptives

${IF}_{k}^{I}$ = HIV infected women not using contraceptives, k = 1..5

${IF}^{RX}$ = HIV infected women not using contraceptives receiving ART

SFD = Susceptible women using DMPA

${IFD}_{k}^{I}$ = HIV infected women using DMPA, k = 1..5

${IFD}^{RX}$ = HIV infected women using DMPA receiving ART

${SFD}^{P}$ = Susceptible women using DMPA on long-acting PrEP

${IFD}_{k}^{p}$ = HIV infected women using DMPA, infected despite use of long-acting PrEP, k = 1..5

SFC = Susceptible women using other contraceptives

${IFC}_{k}^{I}$ = HIV infected women using other contraceptives, k = 1..5

${IFC}^{RX}$ = HIV infected women using other contraceptives receiving ART

${SFC}^{P}$ = Susceptible women using other contraceptives on long-acting PrEP

${IFC}_{k}^{p}$ = HIV infected women using other contraceptives, infected despite use of long-acting PrEP, k = 1..5

*Other variables*

$\lambda$M = Force of infection for men

$\lambda$F = Force of infection for women

${\lambda F}^{p}$ = Force of infection when using other contraceptives with long-acting PrEP

μ = Mortality general population

μ_k_ = Mortality untreated HIV infected patients in infection stage *k*, k = 1..5

μ^RX^ = Mortality treated patients

γ_k_ = HIV infection progression rate by stage *k*, k = 1..4

α = HIV test rate in people using long-acting PrEP

τ = Proportion long-acting PrEP users starting treatment after being tested HIV positive

RX_k_ = Proportion HIV infected individuals in stage *k* who start treatment, k = 1..5

d = Proportion HIV positive women who start DMPA

d^S^ = Proportion HIV negative women who start DMPA

d_off_ = Proportion HIV positive DMPA users who discontinue DMPA

$d_{off}^{S}$ = Proportion HIV negative women who discontinue DMPA

d^p^ = Proportion women who start DMPA and long-acting PrEP

$d_{off}^{p}$ = Proportion women who discontinue DMPA and long-acting PrEP

θ = Proportion women who start other contraceptives

θ_off_ = Proportion women who discontinue other contraceptives

θ^p^ = Proportion women who start other contraceptives and long-acting PrEP

${}_{off}^{p}$ = Proportion women who discontinue other contraceptives and long-acting PrEP

ω^d^ = Proportion DMPA users who start using long-acting PrEP

${}_{Off}^{d}$ = Proportion DMPA and long-acting PrEP users who discontinue long-acting PrEP

ω^c^ = Proportion other contraceptive users who start using long-acting PrEP

${}_{Off}^{c}$ = Proportion other contraceptive and long-acting PrEP users who discontinue long-acting PrEP

ν = Proportion other contraceptive users who switch to DMPA with long-acting PrEP

π = Proportion DMPA and long-acting PrEP users who switch to other contraceptives without long-acting PrEP

Ν = Proportion DMPA users who switch to other contraceptives

Ν_P_ = Proportion DMPA and long-acting PrEP users who switch to other contraceptives with long-acting PrEP

Π = Proportion other contraceptive users who switch to DMPA

Π_P_ = Proportion other contraceptive with long-acting PrEP users who switch to DMPA with long-acting PrEP

Ψ = Population growth (new 15 years old per year)

N_Men_ = Total population of men

N_Women_ = Total population of women

*Table S1: number of sexual partnerships per year for different periods in time*

The number of sexual partnerships per year (*Ω)* is calibrated in the model. To mimic the South African epidemic, this number was higher in the beginning of the epidemic but decreased due to behavioural changes. [3]

| **Year** | **Range** |
| --- | --- |
| Until 1995 | 1.75 – 3.24 |
| 1995-2000 | 0.88 – 1.62 |
| After 2001 | 0.53 – 0.97 |

Ordinary Differential Equations

*Men*

[1] SM = $\frac{1}{2}$Ψ – SM$\lambda$M – SMμ

[2] ${IM}_{1}^{I}$= SM$\lambda$M – ${IM}_{1}^{I}$(γ_1_ + RX_1_ + μ_1_)

[3] ${IM}_{k}^{I}$= γ_k-1_${IM}_{k-1}^{I}$ – ${IM}_{k}^{I}$(γ_k_ + RX_k_ + μ_k_) k = 2..4

[4] ${IM}_{5}^{I}$= γ_4_${IM}_{4}^{I}$ – ${IM}_{5}^{I}$(RX_k_ + μ_k_)

[5] ${IM}^{RX}$=$\sum_{k=1}^{5} {IM}_{k}^{I}$RX_k_ – IM^RX^μ^RX^

*Women without contraceptives*

[6] SF = $\frac{1}{2}$Ψ + SFD$d_{off}^{S}$ + SFD^p^$d_{off}^{p}$ + SFCθ_off_ + SFC^P^${}_{off}^{p}$ – SF($\lambda$F + d^S^ + d^p^ + θ + θ^p^ + μ)

[7] ${IF}_{1}^{I}$= SF$\lambda$F + ${IFD}_{1}^{I}$d_off_ + ${IFD}_{1}^{p}d_{off}^{p}$ + ${IFC}_{1}^{I}$θ_off_ +${IFC}_{1}^{p}{}_{off}^{p}$ – ${IF}_{1}^{I}$(γ_1_+ d + θ + RX_1_ + μ_1_)

[8] ${IF}_{k}^{I}$= γ_k-1_${IF}_{k-1}^{I}$+ ${IFD}_{k}^{I}$d_off_ + ${IFD}_{k}^{p}d_{off}^{p}$ + ${IFC}_{k}^{I}$θ_off_ +${IFC}_{k}^{p}{}_{off}^{p}$ – ${IF}_{k}^{I}$(γ_k_ + d + θ + RX_k_ +μ_k_) k = 2..4

[9] ${IF}_{5}^{I}$= γ_4_${IF}_{4}^{I}$+ ${IFD}_{5}^{I}$d_off_ + ${IFC}_{5}^{I}$θ_off_  – ${IF}_{k}^{I}$(d + θ + RX_5_ + μ_5_)

[10] ${IF}^{RX}$= $\sum_{k=1}^{5} {IF}_{k}^{I}$RX_k_ + IFD^RX^d_off_ + IFC^RX^θ_off_ – ${IF}^{RX}$(d + θ + μ^RX^)

*Women using DMPA without long-acting PrEP*

[11] SFD = SFd^S^ + ${SFD}^{P}{}_{Off}^{d}$ + SFCΠ – SFD($\lambda$D + $d_{off}^{S}$ + ω^d^ + Ν + μ)

[12] ${IFD}_{1}^{I}$= SFD$\lambda$D + ${IFD}_{1}^{p}$(1-τ)α +${IF}_{1}^{I}$d + ${IFC}_{1}^{I}$Π – ${IFD}_{1}^{I}$(γ_1_+ d_off_ + Ν + RX_1_ + μ_1_)

[13] ${IFD}_{k}^{I}$= γ_k-1_${IFD}_{k-1}^{I}$+ ${IFD}_{k}^{p}$(1-τ)α + ${IF}_{k}^{I}d$ + ${IFC}_{k}^{I}$Π – ${IFD}_{k}^{I}$(γ_k_ + d_off_ +Ν + RX_k_ +μ_k_) k = 2..4

[14] ${IFD}_{5}^{I}$= γ_4_${IFD}_{4}^{I}$+ ${IF}_{5}^{I}d$ + ${IFC}_{5}^{I}$Π – ${IFD}_{5}^{I}$(d_off_ +Ν + RX_k_ + μ_5_)

[15] ${IFD}^{RX}$= $\sum_{k=1}^{5} {IFD}_{k}^{I}$RX_k_ + $\sum_{k=1}^{4} {IFD}_{k}^{P}$τα + ${IFD}_{5}^{P}$α + IF^RX^d + IFC^RX^Π – ${IFD}^{RX}$(d_off_ + Ν + μ^RX^)

*Women using DMPA with long-acting PrEP*

*[16]* ${SFD}^{P}$= SFd^p^ + SFDω^d^ + SFC^P^Π_p_ + SFCν – SFD^P^($\lambda$D^p^ + $d_{off}^{p}$+ ${}_{Off}^{d}$+ π + Ν_P_ + μ)

[17] ${IFD}_{1}^{p}$= SFD^p^$\lambda$D^p^ + ${IFC}_{1}^{p}$Π_p_ – ${IFD}_{1}^{p}$(γ_1_ + $d_{off}^{p}$+ π + Ν_P_ + τα + (1-τ)α + μ_1_)

[18] ${IFD}_{k}^{p}$= γ_k-1_${IFD}_{k-1}^{p}$+ ${IFC}_{k}^{p}$Π_p_ – ${IFD}_{k}^{p}$(γ_k_ + $d_{off}^{p}$+ π + Ν_P_ + τα + (1-τ)α + μ_k_) k = 2..4

[19] ${IFD}_{5}^{p}$= γ_4_${IFD}_{4}^{p}$+ ${IFC}_{k}^{p}$Π_p_ – ${IFD}_{5}^{p}$(π + Ν_P_ + α + μ_5_)

*Women using other contraceptives without long-acting PrEP*

[20] SFC = SFθ + SFC^p^${}_{Off}^{c}$+ SFDΝ + SFD^P^N_P_ – SFC($\lambda$F + θ_off_ + ω^c^ + Π + ν + μ)

[21] ${IFC}_{1}^{I}$= SFC$\lambda$F + ${IFC}_{1}^{p}$(1-τ)α + ${IF}_{1}^{I}$ + ${IFD}_{1}^{I}$Ν + ${IFD}_{1}^{P}$π – ${IFC}_{1}^{I}$(γ_1_+ θ_off_ + Π + RX_1_ +μ_1_)

[22] ${IFC}_{k}^{I}$= γ_k-1_${IFC}_{k-1}^{I}$+${IFC}_{k}^{p}$(1-τ)α +${IF}_{k}^{I}$ + ${IFD}_{k}^{I}$Ν +${IFD}_{k}^{p}$π – ${IFC}_{k}^{I}$(γ_k_ + θ_off_ + Π + RX_k_ + μ_k_) k = 2..4

[23] ${IFC}_{5}^{I}$= γ_4_${IFC}_{4}^{I}$ + ${IF}_{5}^{I}$ + ${IFD}_{5}^{I}$Ν + ${IFD}_{5}^{p}$π – ${IFC}_{5}^{I}$(θ_off_ + Π + RX_5_ + μ_5_)

[24] ${IFC}^{RX}$= $\sum_{k=1}^{5} {IFC}_{k}^{I}$RX_k_ + $\sum_{k=1}^{4} {IFC}_{k}^{P}$τα + ${IFC}_{5}^{P}$α + IF^RX^θ + IFD^RX^Ν – ${IFC}^{RX}$(θ_off_ + Π + μ^RX^)

*Women using other contraceptives with long-acting PrEP*

[25] ${SFC}^{P}$= SFθ^p^ + SFCω^c^ + SFD^P^Ν_P_ – SFC^P^($\lambda$F^P^ + ${}_{off}^{p}$ + ${}_{Off}^{c}$ + Π_P_ + μ)

[26] ${IFC}_{1}^{p}$= ${SFC}^{P}\lambda$F^P^ + ${IFD}_{1}^{p}$Ν_P_ – ${IFC}_{1}^{P}$(γ_1_+ Π_P_ + ${}_{off}^{p}$ + τα + (1- τ)α + μ_1_)

[27] ${IFC}_{k}^{p}$= γ_k-1_${IFC}_{k-1}^{P}$+ ${IFD}_{k}^{p}$N_P_ – ${IFC}_{k}^{p}$(γ_k_ + Π_P_ + ${}_{off}^{p}$ + τα + (1- τ)α + μ_k_) k = 2..4

[28] ${IFC}_{5}^{p}$= γ_4_${IFC}_{4}^{P}$+ ${IFD}_{5}^{p}$N_P_ – ${IFC}_{5}^{p}$(Π_P_ + α + μ_5_)

*Population size*

[29] N_Men_ = SM + $\sum_{k=1}^{5} {IM}_{k}^{I}$+ ${IM}^{RX}$

[30] N_Women_ = SF + SFD + SFC + SFD^P^ + SFC^P^ + $\sum_{k=1}^{5} {IF}_{k}^{I}$+ $\sum_{k=1}^{5} {IFD}_{k}^{I}$+ $\sum_{k=1}^{5} {IFD}_{k}^{P}$+ $\sum_{k=1}^{5} {IFC}_{k}^{P}$+ $\sum_{k=1}^{5} {IFC}_{k}^{I}$+ ${IF}^{RX}$ + ${IFD}^{RX}$ + ${IFC}^{RX}$

*Force of infection*

The forces of infection ($\lambda$) for the different population groups are given below. They describe the rate by which susceptible individuals become HIV infected. This is determined by the number of sexual partnerships per year (*Ω)* and the infectiousness of individuals in each class (β). The infectiousness differs per HIV stage (table 1), where β_K_ and β_RX_ describe the infectiousness in the HIV stages *k* and the infectiousness while using ART. Women have a higher risk of becoming HIV infected compared to men (*κ).*The use of long-acting PrEP protects against HIV infection, *φ* is the effectiveness of long-acting PrEP. In this model, the infectiousness of women who become HIV infected despite the use of long-acting PrEP is lowered with the effectiveness of long-acting PrEP.

[31.] $\lambda$M =

$\frac{(\sum_{k=1}^{5} {IF}_{k}^{I}K+\sum_{k=1}^{5} {IFD}_{k}^{I}K+\sum_{k=1}^{5} {IFC}_{k}^{I}K+(\sum_{k=1}^{5} {IFD}_{k}^{P}K+\sum_{k=1}^{5} {IFC}_{k}^{P}K) (1-)+({IF}^{RX}+ {IFD}^{RX} + {IFC}^{RX}) \mathrm{RX}}{N_{Women}}$

[32] $\lambda$F = $\frac{(\sum_{K=1}^{5} {IM}_{k}^{I}\beta_{k}+ {IM}^{RX}\beta_{RX})}{N_{Men}}$

[34] ${\lambda F}^{p}$ = $\frac{(1-)(\sum_{K=1}^{5} {IM}_{k}^{I}\beta_{k}+ {IM}^{RX}\beta_{RX})}{N_{Men}}$

*Table S2A: calibrated variables for the use of different contraceptive methods in the period before scale-up of long-acting pre-exposure prophylaxis [PrEP]*

| **Variable** | **Range** |
| --- | --- |
| d | 0.020 – 0.12 |
| d^S^ | 0.020 – 0.12 |
| d_off_ | 0.20 – 0.55 |
| $d_{off}^{S}$ | 0.20 – 0.55 |
| θ | 0.10 – 0.44 |
| θ_off_ | 0.10 – 0.30 |
| Ν | 0.040 – 0.19 |
| Π | 0.040 – 0.19 |

*Table S2B Variables after scale-up period of long-acting pre-exposure prophylaxis [PrEP]. Values are adapted to obtain calibrated proportions of injectable contraceptive users on long-acting PrEP.*

| **Percentage injectable contraceptive users on long-acting PrEP** | | |  |
| --- | --- | --- | --- |
| **25%** | **50%** | **75%** |  |
| **d** | 0.020 – 0.12 | 0.020 – 0.12 | 0.020 – 0.12 |
| **d^S^** | 0.014 – 0.084 | 0.010 – 0.06 | 0.011 – 0.066 |
| **d_off_** | 0.20 – 0.55 | 0.20 – 0.55 | 0.20 – 0.55 |
| $\boldsymbol{d}_{\boldsymbol{off}}^{\boldsymbol{S}}$ | 0.15– 0.41 | 0.30 – 0.83 | 0.44 – 1.21 |
| **θ** | 0.066 – 0.29 | 0.063 – 0.28 | 0.056 – 0.25 |
| **θ_off_** | 0.10 – 0.30 | 0.10 – 0.30 | 0.10 – 0.30 |
| **Ν** | 0.030 – 0.14 | 0.020 – 0.10 | 0.080 – 0.38 |
| **Π** | 0.030 – 0.14 | 0.020 – 0.10 | 0.040 – 0.19 |
| **d^p^** | 0.0050 – 0.03 | 0.10 – 0.28 | 0.016 – 0.096 |
| $\boldsymbol{d}_{\boldsymbol{off}}^{\boldsymbol{p}}$ | 0.18 – 0.50 | 0.10 – 0.28 | 0.20 – 0.55 |
| **θ^p^** | 0.0054 – 0.024 | 0.006 – 0.028 | 0.015 – 0.066 |
| ${}_{\boldsymbol{off}}^{\boldsymbol{p}}$ | 0.10 – 0.30 | 0.10 – 0.30 | 0.10 – 0.30 |
| **Π_P_** | 0.020 – 0.095 | 0.020 – 0.10 | 0.030 – 0.14 |
| **ν** | 0.010 – 0.048 | 0.05 – 0.15 | 0.040 – 0.19 |
| **Ν_P_** | 0.020 – 0.10 | 0.02 – 0.10 | 0.030 – 0.14 |
| **π** | 0.010 – 0.048 | 0.02 – 0.10 | 0.040 – 0.19 |
| **ω^d^** | 0.050 – 0.14 | 0.10 – 0.28 | 0.24 – 0.66 |
| **ω^c^** | 0.0055 – 0.026 | 0.011 – 0.052 | 0.022 – 0.10 |
| ${}_{\boldsymbol{Off}}^{\boldsymbol{d}}$ | 0.12 – 0.33 | 0.10 – 0.28 | 0.050 – 0.14 |
| ${}_{\boldsymbol{Off}}^{\boldsymbol{c}}$ | 0.058 – 0.27 | 0.050 – 0.23 | 0.040 – 0.19 |

**Treatment guidelines through the years**

Before 2003, access to antiretroviral treatment in resource limited settings was very low. Therefore, in our model HIV treatment becomes available for patients in the AIDS stage as of 2003.[4] In 2010, treatment was initiated for infected individuals with a CD4<350 cells per μl [5] and in 2013 this threshold was expanded to infected individuals with CD4<500 cells per μl [6]. The current treatment guidelines are from 2015 and recommend HIV treatment for all HIV infected individuals.[7] So, in our model treatment for chronically infected individuals with CD4>500 cells per μl or individuals in the acute HIV stage becomes available as of 2015.

*Table S3: Range of values used for rates of getting treatment after 2017*

The rate of starting treatment before 2018 will result in an overall proportion of 85% of HIV infected individuals that will be using treatment in 2030. The HIV treatment rates in 2018 and after were varied equally for all disease stages to obtain 75% or 95% of HIV-infected individuals using ART in 2030.

| **Proportion infected individuals using ART in 2030** | | |  |
| --- | --- | --- | --- |
| **75%** | **85%** | **95%** |  |
| **Acute HIV** | 0.00 – 0.036 | 0.00 – 0.099 | 0.00 – 0.20 |
| **Chronic HIV CD4 > 500 cells per μL** | 0.019 – 0.096 | 0.042 – 0.27 | 0.10 – 0.56 |
| **Chronic HIV CD4 350 – 500 cells per μL** | 0.019 – 0.096 | 0.042 – 0.27 | 0.10 – 0.56 |
| **Chronic HIV CD4 200-350 cells per μL** | 0.08 – 0.31 | 0.18 – 0.70 | 0.44 – 1.40 |
| **AIDS** | 0.12 – 0.31 | 0.27 – 0.85 | 0.67 – 1.70 |

**Assessing the cost-effectiveness**

**Disability-adjusted life-years (DALYs)**

All DALYs are discounted with 3% per year.

*Table S4: Assumed disability weightings for DALYs.*

| **Status** | **Utility Weight** | **Source** |
| --- | --- | --- |
| Susceptible/On long-acting PrEP | 0* | [8], assumption |
| Acutely infected | 0.051** | [8] |
| Chronically infected CD4>500 cells/μL | 0.051** | [8] |
| Chronically infected CD4 350-500 cells/μL | 0.051** | [8] |
| Chronically infected CD4 200-350 cells/μL | 0.221 | [8] |
| Infected AIDS stage | 0.547 | [8] |
| Infected on treatment | 0.053** | [8](Tengs, 2002 #125;Vos T, 2015 #153) |

* Side effects of long-acting PrEP are not fully known as long-acting PrEP is currently under development. So far, reported side effects have been mild and transient. However, if a disability weight of 0.0005 is assumed when using long-acting PrEP to account for potential side effects of long-acting PrEP, comparable to disability weights used in [9, 10], our baseline scenario would cost $1681/DALY averted compared to $1657/DALY averted if no adjustment in utility weight is considered for long-acting PrEP users.

**These utility weights were used by previous studies, e.g. [11, 12], however other studies also considered the same utility weight for individuals on treatment as compared to individuals who are acutely or chronically infected with a CD4 cell count >350/μL. If we assume the utility weight when acutely infected or chronically infected with CD4>350/μL are also 0.053 comparable to [13], our baseline scenario would cost $1694/DALY averted compared to $1657/DALY averted, so we trust this does not affect our outcomes.

**Costs**

Included costs are: costs for antiretroviral therapy (ART), costs for long-acting pre-exposure prophylaxis *[PrEP]* and costs for negative and positive HIV tests in the population. The yearly HIV test-rate in the susceptible population is every three years.[14] Costs for positive HIV tests are assessed by one-off costs for individuals newly starting ART. All costs are discounted with 3% per year. Costs for ART and drug cost of long-acting PrEP are varied in a sensitivity analysis.

*Table S5A: Costs for women using long-acting pre-exposure prophylaxis [PrEP] in Limpopo [15]*

| **Average costs long-acting PrEP** [15] | **First year ($)** | **Every year thereafter ($)** |
| --- | --- | --- |
| Drugs | unknown | unknown |
| Labs | 35.22 | 17.02 |
| HIV testing services | 7.83 | 7.56 |
| Screening and treatment STI | 2.83 | 2.53 |
| Social mobilization | 8.25 | 8.25 |
| Counselling/dispensing | 10.40 | 10.10 |
| Combination prevention | 2.50 | 2.43 |
| Overheads | 11.20 | 10.00 |
| Training | 4.51 | 4.51 |
| Monitoring and evaluation of the program | 0.43 | 0.43 |
| **Total excluding drug costs*** | 83.17 | 62.84 |

*We based long-acting PrEP costs on an average use of five years. Yearly costs per individual using long-acting PrEP is therefore $66.91 (excluding drug costs).

STI = Sexually transmitted infections

In our sensitivity analysis, we assume non-drug costs of long-acting PrEP could decrease by 30% if long-acting PrEP could be administered in the same healthcare visit as the injectable contraceptives.

*Table S5B: Overview used costs*

|  | **Costs ($)** | **Source** |
| --- | --- | --- |
| Negative HIV test | $4.22 | [15] |
| Positive HIV test | $6.22 | [15] |
| Yearly treatment with ART | $249.52 | [15] |
| Yearly treatment with long-acting PrEP excluding drug costs | $66.91 | [15] |

ART = anti-retroviral therapy, PrEP = pre-exposure prophylaxis

*Table S6: Variables used to calibrate and accept simulations using Monte Carlo filtering techniques.* 348 simulations were accepted from 490,000 total simulations run.

| **Parameter** | **Reference value** | **Accepted values** | **Source** |
| --- | --- | --- | --- |
| *Population Size Limpopo (15-49 years old)* |  |  |  |
| 2010 | 2 852 900 | 2 500 000 – 3 200 000 | [16] |
| 2011 | 2 894 859 | 2 550 000 – 3 300 000 | [17] |
| 2012 | - | 2 500 000 – 3 350 000 |  |
| 2013 | 2 985 853 | 2 650 000 – 3 350 000 | [18] |
| 2014 | 2 974 642 | 2 650 000 – 3 350 000 | [19] |
| 2015 | 3 061 837 | 2 700 000 – 3 400 000 | [20] |
| 2016 | 3 155 819 | 2 800 000 – 3 500 000 | [21] |
| 2017 | 2 960 294 | 2 650 000 – 3 350 000 | [22] |
| *HIV prevalence women*  *(15-49 years old)* |  |  | [14] |
| 2000 | 8.1% | 6.1% – 10.1% |  |
| 2005 | 12.5% | 10.5% – 14.5% |  |
| 2010 | 15.6% | 13.6% – 17.6% |  |
| 2011 | 16.0% | 14.0% – 18.0% |  |
| 2012 | 16.3% | 14.3% – 18.3% |  |
| 2013 | 16.7% | 14.7% – 18.7% |  |
| 2014 | 17.0% | 15.0% – 19.0% |  |
| 2015 | 17.2% | 15.2% – 19.2% |  |
| 2016 | 17.5% | 15.5% – 19.5% |  |
| 2017 | 17.6% | 15.6% – 19.6% |  |
| *HIV prevalence men*  *(15-49 years old)* |  |  | [14] |
| 2000 | 4.5% | 2.5% – 6.5% |  |
| 2005 | 6.6% | 4.6% – 8.6% |  |
| 2010 | 7.7% | 5.7% – 9.7% |  |
| 2011 | 7.9% | 5.9% – 9.9% |  |
| 2012 | 8.0% | 6.0% – 10.0% |  |
| 2013 | 8.1% | 6.1% – 10.1% |  |
| 2014 | 8.2% | 6.2% – 10.2% |  |
| 2015 | 8.3% | 6.3% – 10.3% |  |
| 2016 | 8.3% | 6.3% – 10.3% |  |
| 2017 | 8.3% | 6.3% – 10.3% |  |
| *HIV-infected individuals on ART in Limpopo* |  |  | [14] |
| 2004 | 0.8% | 0.05% – 5.8% |  |
| 2008 | 10.1% | 2.0% – 20.1% |  |
| 2012 | 36.7% | 26.7% – 46.7% |  |
| 2013 | 42.4% | 32.4% – 52.4% |  |
| 2014 | 48.3% | 38.3% – 58.3% |  |
| 2015 | 54.3% | 44.3% – 64.3% |  |
| 2016 | 60.3% | 50.3% – 70.3% |  |
| 2017 | 66.0% | 60.0% – 72.6% |  |
| Use of DMPA in 2016 | 12.2% | 8.2% – 16.2% | [23] |
| Use of other contraceptives in 2016 | 38.5% | 28.5% – 48.5% | [23] |

DMPA = depot medroxyprogesterone acetate

*Figure S2. The model is calibrated to (A) The population size in Limpopo (B) Proportion HIV-infected individuals (15-49 years) using antiretroviral treatment (ART) in Limpopo (C) HIV prevalence in men (15-49 years) in Limpopo and (D) HIV prevalence in women (15-49 years) in Limpopo.*

The HIV prevalence in men and women and the proportion of HIV-infected individuals using ART are calibrated to data from the Thembisa model which is based on historic data. Depicted are the median (black line) with the minimum and maximum values of all accepted simulations in orange.

**

*Figure S3: Epidemiological impact of long-acting pre-exposure prophylaxis [PrEP] with an effectiveness of 75% on the HIV epidemic in Limpopo, assuming 50% of injectable contraceptives users use long-acting PrEP and 85% of infected individuals uses antiretroviral therapy (ART) by 2030. Total prevented infections and infections prevented separated by men and women are depicted. Prevented infections in men are an indirect result of long-acting PrEP use by women. Depicted are the median and interquartile ranges of all accepted simulations.*

*Figure S4. Effects of the coverage with antiretroviral therapy [23] in the population and long-acting pre-exposure prophylaxis [PrEP] targeted to injectable contraceptive users in Limpopo on the number of new HIV infections in the period 2018-2030. Baseline scenario assumes no long-acting PrEP and 85% of infected individuals using ART by 2030. Effects of lower (75%) and higher (95%) ART coverage in 2030 are depicted as well as the effect of long-acting PrEP for all three different ART scenarios. (A) 25% of HIV-negative injectable users use long-acting PrEP (B) 75% of HIV-negative injectable users use long-acting PrEP. Depicted are the median and interquartile ranges of all accepted simulations.*

*LA-PrEP = long-acting pre-exposure prophylaxis*

*Figure S5. Cost-effectiveness of providing half of HIV negative injectable contraceptive users in Limpopo with long-acting pre-exposure prophylaxis (PrEP.) if the proportion of HIV infected individuals using antiretroviral therapy (ART) a) increases to 75% by 2030; b) increases as predicted to 85% by 2030; c) increases to 95% by 2030. A time horizon of 40 years is used. Red represents scenarios not cost-effective (costs over $1119/DALY), light green represents potentially cost-effective scenarios (cost between $519–$1119 per DALY) and dark green represents cost-effective scenarios (cost <$519/DALY). To be considered (potentially) cost-effective, at least 90% of accepted simulations have an incremental cost-effectiveness ratio below the (potentially) cost-effectiveness threshold.*

**Budget impact**

We estimated the total undiscounted costs due to long-acting PrEP use by half of HIV negative injectable contraceptive users in Limpopo, for time horizons between one to five years after full scale up. Yearly drug prices of long-acting PrEP were varied between $0 and $100 per person and a scenario with the maximum drug price where long-acting PrEP would be potentially cost-effective [$16] was included.

*Table S6: Budget impact of providing half of HIV negative injectable contraceptive users with long-acting PrEP, assuming our baseline scenario with 85% ART coverage in 2030 and long-acting PrEP effectiveness of 75%. Program costs are depicted in million dollars. Drug costs of long-acting PrEP are ranged between $0-$100 per person per year, including $16, the maximum drug price for which long-acting PrEP was found to be potentially cost-effective. Non-drug related costs are $66.91, see Table S5A. Time horizons range between one to five years after full scale up. PrEP = Pre-exposure prophylaxes.*

| Drug price long-acting PrEP | 1 year | 2 years | 3 years | 4 years | 5 years |
| --- | --- | --- | --- | --- | --- |
| $100 | 25.1 | 51.2 | 77.3 | 103.9 | 130.5 |
| $80 | 22.1 | 45.0 | 68.1 | 91.4 | 114.8 |
| $60 | 19.1 | 38.9 | 58.8 | 78.8 | 99.1 |
| $40 | 16.1 | 32.7 | 49.5 | 66.3 | 83.4 |
| $20 | 13.1 | 26.6 | 40.2 | 53.8 | 67.7 |
| $16 | 12.5 | 25.4 | 38.4 | 51.3 | 64.6 |
| $0 | 10.1 | 20.5 | 30.1 | 41.3 | 52.0 |

**References**

1. Polis, C.B., et al., *An updated systematic review of epidemiological evidence on hormonal contraceptive methods and HIV acquisition in women.* Aids, 2016. **30**(17): p. 2665-2683.

2. Ahmed K, B.J., Beksinska M, Bekker L‐G, Bukusi EA, Donnell D, et al, *HIV incidence among women using intramuscular depot medroxyprogesterone acetate, a copper intrauterine device, or a levonorgestrel implant for contraception: a randomised, multicentre, open‐label trial.* Lancet, 2019. **394:303–13. 10.1016/S0140-6736(19)31288-7**.

3. Hallett, T.B., et al., *Declines in HIV prevalence can be associated with changing sexual behaviour in Uganda, urban Kenya, Zimbabwe, and urban Haiti.* Sex Transm Infect, 2006. **82 Suppl 1**: p. i1-8.

4. WHO, *Scaling up antiretroviral therapy in resource-limited settings: treatment guidelines for a public health approach*. 2003, World Health Organization: Geneva.

5. WHO, *Antiretroviral therapy for HIV infection in adults and adolescents - Recommendations for a public health approach 2010 revision*. 2010, WHO Library Cataloguing-in-Publication Data.

6. WHO, *Consolidated guidelines on the use of antiretroviral drugs for treating and preventing HIV infection: recommendations for a public health approach*, J. 2013, Editor. 2013, World Health Organization: Geneva.

7. WHO, *Guideline on when to start antiretroviral therapy and on pre-exposure prophylaxis for HIV*. 2015, WHO Library Cataloguing-in-Publication Data.

8. Vos T, B.R., Bell B, Bertozzi-Villa A, Biryukov S, Bolliger I, et al, *Global, regional, and national incidence, prevalence, and years lived with disability for 301 acute and chronic diseases and injuries in 188 countries, 1990–2013: a systematic analysis for the Global Burden of Disease Study 2013.* The Lancet, 2015. **386(9995):743-800.**

9. Nichols, B.E., et al., *Cost-effectiveness of pre-exposure prophylaxis (PrEP) in preventing HIV-1 infections in rural Zambia: a modeling study.* PLoS One, 2013. **8**(3): p. e59549.

10. Nichols, B.E., et al., *Cost-effectiveness of PrEP in HIV/AIDS control in Zambia: a stochastic league approach.* J Acquir Immune Defic Syndr, 2014. **66**(2): p. 221-8.

11. Quaife, M., et al., *The cost-effectiveness of multi-purpose HIV and pregnancy prevention technologies in South Africa.* J Int AIDS Soc, 2018. **21**(3).

12. Jewell, B.L., et al., *Estimating the cost-effectiveness of pre-exposure prophylaxis to reduce HIV-1 and HSV-2 incidence in HIV-serodiscordant couples in South Africa.* PLoS One, 2015. **10**(1): p. e0115511.

13. Eaton, J.W., et al., *Health benefits, costs, and cost-effectiveness of earlier eligibility for adult antiretroviral therapy and expanded treatment coverage: a combined analysis of 12 mathematical models.* Lancet Glob Health, 2014. **2**(1): p. e23-34.

14. Johnson LF, e.a., *Progress towards the 2020 targets for HIV diagnosis and antiretroviral treatment in South Africa.* Southern African Journal of HIV Medicine, 2016. **2017; 18(1): a694.**

15. Dyer, O., *New 20 year patents threaten to end AIDS drugs for developing countries.* BMJ, 2004. **329**(7478): p. 1308-130c.

16. *Mid-year population estimates 2010*, in *Statistical release P0302*. 2010, Statistics South Africa: Pretoria, South Africa.

17. *Mid-year population estimates 2011*, in *Statistical release P0302*. 2011, Statistics South Africa: Pretoria South Africa.

18. *Mid-year population estimates 2013*, in *Statistical release P0302*. 2013, Statistics South Africa: Pretoria, South Africa.

19. *Mid-year population estimates 2014*, in *Statistical release P0302*. 2014, Statistics South Africa: Pretoria, South Africa.

20. *Mid-year population estimates 2015*, in *Statistical release P0302*. 2015, Statistics South Africa: Pretoria, South Africa.

21. *Mid-year population estimates 2016*, in *Statistical release P0302*. 2016, Statistics South Africa: Pretoria South Africa.

22. *Mid-year population estimates 2017*, in *statistical release P0302*. 2017, Statistics South Africa: Pretoria, South Africa.

23. Carobene, M.G., et al., *Differences in frequencies of drug resistance-associated mutations in the HIV-1 pol gene of B subtype and BF intersubtype recombinant samples.* J.Acquir.Immune.Defic.Syndr., 2004. **35**(2): p. 207-209.
